# The Interface of Oral and Brain Health: Current Insights Into the Bidirectional Relationship Between Alzheimer's Disease and Periodontitis

**DOI:** 10.1002/cns.70754

**Published:** 2026-01-26

**Authors:** Weidong Zhang, Shasha Li, Yajun Cui, Yuhui Lun, Hongrui Liu, Minqi Li

**Affiliations:** ^1^ Department of Bone Metabolism, School and Hospital of Stomatology, Cheeloo College of Medicine Shandong University & Shandong Key Laboratory of Oral Tissue Regeneration & Shandong Engineering Research Center of Dental Materials and Oral Tissue Regeneration & Shandong Provincial Clinical Research Center for Oral Diseases Jinan China; ^2^ Center of Osteoporosis and Bone Mineral Research Shandong University Jinan China; ^3^ Department of Pediatrics Dentistry Binzhou Medical University Hospital Binzhou China; ^4^ Department of Stomatology Binzhou People's Hospital Binzhou China; ^5^ Department of Periodontology, School and Hospital of Stomatology Cheeloo College of Medicine, Shandong University Jinan China

**Keywords:** aging, Alzheimer's disease, bone remodeling, neuroinflammation, oral‐brain axis, periodontitis

## Abstract

**Background:**

The bidirectional relationship between Alzheimer's disease (AD) and periodontitis highlights a critical interface between oral and brain health, increasingly recognized as a key axis in aging‐related degenerative diseases. This review synthesizes epidemiological, pathological, and mechanistic evidence underpinning their interconnected pathogenesis.

**Results and Conclusion:**

Epidemiologically, periodontitis is associated with an increased risk of AD, while AD patients exhibit a higher prevalence of periodontitis, driven by cognitive decline‐impaired oral hygiene and synergistic inflammatory mechanisms. Pathologically, periodontal pathogens (e.g., *Porphyromonas gingivalis* ) translocate to the brain via hematogenous, trigeminal, or oral‐intestinal pathways, inducing neuroinflammation, β‐amyloid (Aβ) aggregation, and tau hyperphosphorylation through virulence factors like gingipains and LPS. Conversely, AD‐related neurodegeneration disrupts bone homeostasis to aggravate periodontitis via multiple mechanisms: (1) Aβ‐mediated osteoclast activation through RAGE/NF‐κB signaling and suppression of osteoblastogenesis; (2) autonomic nervous system dysregulation, where sympathetic hyperactivity promotes RANKL‐dependent bone resorption; and (3) endocrine dysfunction, including HPA axis hyperactivation (chronic hypercortisolism), sex hormone deficiency, insulin resistance, and growth hormone/IGF‐1 insufficiency, all of which perturb the balance between bone formation and resorption. Aging exacerbates this bidirectional interplay through immunosenescence (e.g., impaired neutrophil phagocytosis, senescent microglia) and inflammaging, creating a pro‐degenerative milieu that amplifies both diseases. Clinical interventions, such as periodontal therapy and microbiota modulation, show promise in reducing systemic inflammation and slowing AD progression, though causal links require validation. Future research must prioritize longitudinal cohort studies, pathogen‐specific mechanistic investigations, and translational strategies targeting the oral‐brain axis to develop preventive interventions for aging populations.

## Graphical abstract

Bidirectional Pathogenesis Between AD and Periodontitis. Periodontal pathogens reach the brain via hematogenous/trigeminal/oral–intestinal pathways, triggering neuroinflammation and Aβ plaques to drive AD. Conversely, AD disrupts bone homeostasis (via Aβ, endocrine/autonomic dysregulation) to aggravate periodontitis.

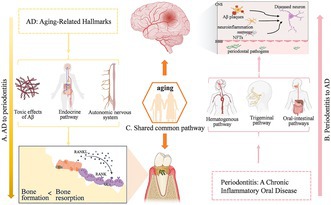

**Abbreviations**
AD: Alzheimer's disease
APP: amyloid precursor protein
Aβ: β‐amyloid
DCs: dendritic cells
GH: growth hormone
GLP‐1: glucagon‐like peptide‐1
GLP‐1: glucagon‐like peptide‐1
GSK‐3β: glycogen synthase kinase‐3β
HPA: hypothalamic–pituitary–adrenal
IGF‐1: growth factor‐1
LTD: enhancing long‐term depression
LTP: long‐term potentiation
NCAM: neural cell adhesion molecule
NETs: Neutrophil extracellular traps
NGF: nerve growth factor
OPG: osteoprotegerin
PNS: parasympathetic nervous system
RANKL: receptor activator of nuclear factor kappa‐B ligand
RCTs: randomized controlled trials
sRAGE: soluble RAGE

## 1 Introduction

Globally, societies are experiencing a profound demographic transition characterized by accelerated population aging, driven by the dual forces of declining fertility rates and unprecedented increases in life expectancy. As reported in the 2022 World Health Organization (WHO) publication, the global population aged 60 years or older is projected to grow from 1 billion to 2.1 billion between 2020 and 2050—representing a twofold increase—while the number of individuals aged 80 years or older is expected to triple, reaching 426 million [1]. The successive arrival of an aging society is accompanied by age‐related diseases. Among them, Alzheimer's disease (AD) and periodontitis, as two high‐incidence diseases, have become key factors affecting the healthy lifespan of the elderly. AD, the most common neurodegenerative disorder, constitutes the primary cause of cognitive impairment in older adults, accounting for 60%–80% of all clinical cases of cognitive decline [2]. Concurrently, periodontal disease—characterized by alveolar bone resorption and chronic gingival inflammation—represents a major etiological factor for tooth loss in older populations [3]. AD is a typical age‐related neurodegenerative disorder, while periodontitis is a chronic infectious inflammatory disease closely related to aging. Although AD and periodontitis exhibit distinct pathological hallmarks, both conditions are tightly regulated by aging‐associated biological processes—including inflammaging, immunosenescence, and oral microbial dysbiosis—and they engage in a bidirectional correlation that amplifies disease progression [4, 5, 6].

Oral health and brain health represent central health indicators in the aging process [7, 8]. The oral cavity functions as a critical “microecological window” to systemic health, where the homeostasis of its microbial community is intimately linked to the body's systemic inflammatory status [9]. Periodontal pathogens and their virulence factors, alongside proinflammatory cytokines, translocate to the central nervous system via hematogenous or trigeminal pathways, inciting neuroinflammation, oxidative stress, and amyloidogenic cascades that elevate the risk for AD and other neurodegenerative disorders [10]. Conversely, brain health—defined by cognitive integrity, synaptic plasticity, and neural network stability—is foundational to maintaining independent function in older adults. Age‐related cognitive decline, particularly in executive and motor domains, impairs oral hygiene compliance (e.g., reduced dexterity for brushing, medication adherence), thereby fostering plaque accumulation and exacerbating periodontal tissue destruction [11]. This review aims to systematically synthesize epidemiological evidence, pathological mechanisms, and advances in clinical intervention research regarding the bidirectional relationship between AD and periodontitis. Specifically, it focuses on delineating the central role of the “oral‐brain axis” in mediating the crosstalk between these two conditions, thereby providing a theoretical framework for the development of interdisciplinary prevention and therapeutic strategies tailored to the elderly population.

## 2 Materials and Methods

### 2.1 Search Strategy

**FIGURE 1:**
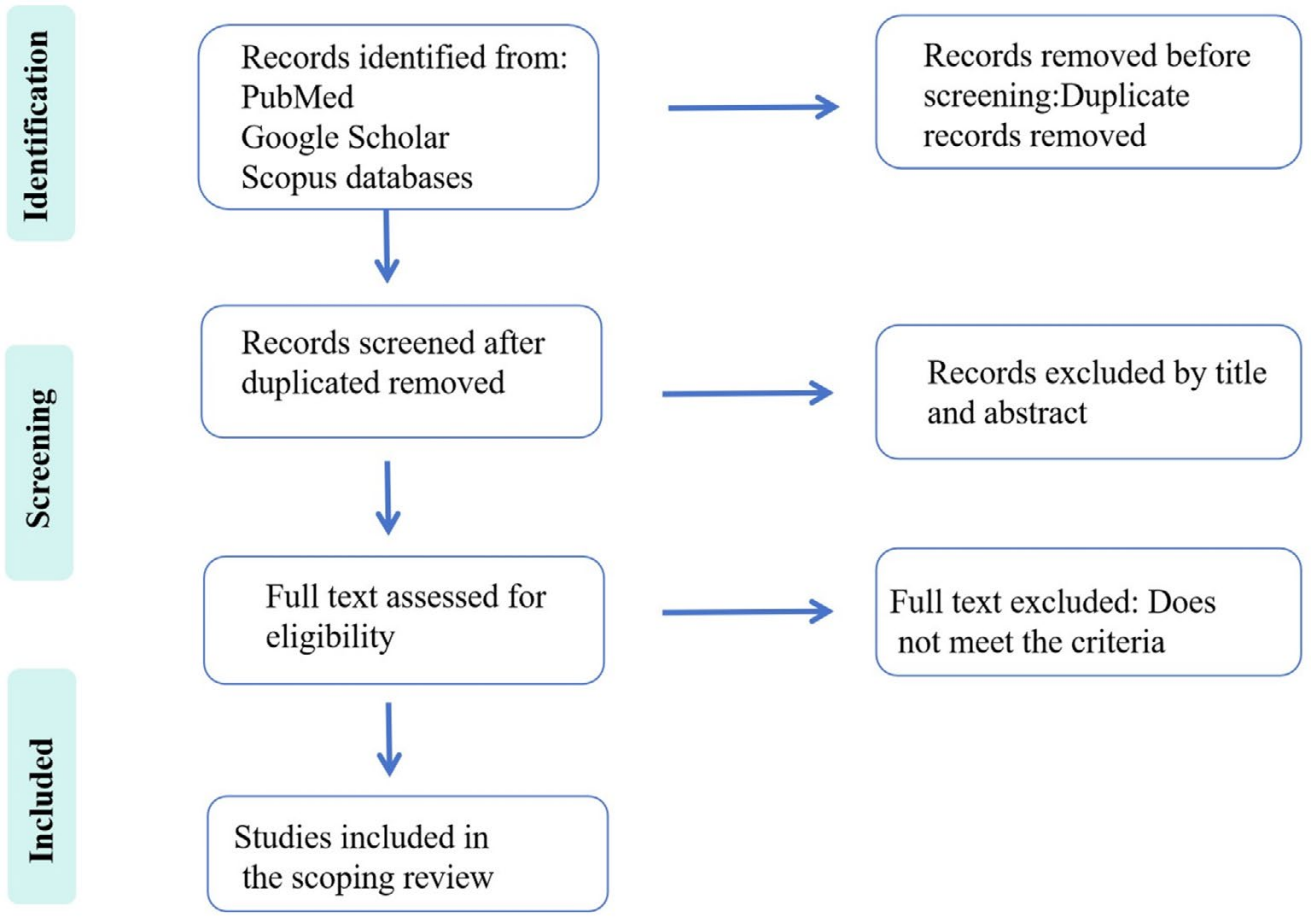
PRISMA flowchart.

Relevant literature was retrieved from the PubMed, Google Scholar, and Scopus databases, covering the publication period from 2015 to 2025. This review included clinical trials and experimental studies conducted on animal models. Additionally, the reference lists of published systematic reviews and meta‐analyses were manually screened to identify additional eligible original studies that were not captured in the initial database searches. The search strategy incorporated the following keywords: “Alzheimer's disease”, “Periodontitis”, “Oral‐brain axis”, “Neuroinflammation”, “Aging”, and “Bone remodeling”. Boolean operators (“AND” and “OR”) were strategically applied to optimize the search strategy, ensuring comprehensive retrieval of studies relevant to the research topic (Figure 1).

### 2.2 Inclusion Criteria

1. Studies exploring the link between periodontitis and Alzheimer's disease (AD), encompassing both direct and indirect mechanistic pathways, were considered for inclusion.
2. Articles focusing on the elderly demographic were prioritized, considering that aging constitutes a major shared risk factor for both conditions.
3. Research that offers clinical, microbiological, or epidemiological data pertinent to the association between these two disorders was included.

### 2.3 Exclusion Criteria

Studies were excluded on the following grounds: failure to meet the predefined inclusion criteria, insufficient methodological rigor, unclear description of research methods, inadequate sample sizes, or poor reproducibility.

## 3 Overview of Alzheimer's Disease and Periodontitis

### 3.1 Alzheimer's Disease: Aging‐Related Hallmarks

AD is characterized by cognitive decline linked to hippocampal and cortical neuronal loss, with pathogenesis centered on three interconnected pillars: β‐amyloid (Aβ) aggregation, tau pathology, and chronic neuroinflammation [12]. Aβ peptides (predominantly Aβ40/42) are generated via aberrant proteolysis of amyloid precursor protein (APP) by β/γ‐secretases [13]; the highly hydrophobic Aβ_42_ readily forms extracellular plaques that impair synaptic plasticity critical for memory [14]. Tau, a microtubule‐stabilizing protein [15], undergoes hyperphosphorylation due to dysregulated kinases (GSK‐3β, CDK5) and reduced phosphatase (PP2A) activity, losing microtubule‐binding capacity and aggregating into intracellular neurofibrillary tangles (NFTs) with prion‐like pathological propagation [16]. Phosphorylated tau can act as a “prion‐like” protein, templating the conversion of native tau into pathological conformations, driving progressive neurodegeneration [17]. Chronic neuroinflammation, driven by dysfunctional microglia and reactive astrocytes, exacerbates neurodegeneration: activated microglia release pro‐inflammatory cytokines (IL‐1β, TNF‐α) and ROS, while reactive astrocytes form gliosis secreting context‐dependent neurotoxic or neuroprotective factors [18]. Though initially protective, sustained neuroinflammation in aging creates a pro‐degenerative microenvironment that amplifies Aβ/tau pathology. Together, these intertwined pathways—dysregulated proteolysis, tau misfolding, and chronic neuroinflammation—form a degenerative loop, exacerbated by aging‐related microglial dysfunction and blood–brain barrier (BBB) impairment, driving progressive synaptic damage and neuronal loss in AD.

### 3.2 Periodontitis: A Chronic Inflammatory Oral Disease

Periodontitis is a prototypical chronic inflammatory oral disease characterized by progressive destruction of periodontal tissues—including the gingiva, periodontal ligament, alveolar bone, and cementum—ultimately leading to tooth loss [19]. Affecting over 10% of the global population, it is marked by deep periodontal pocket formation, gingival inflammation, and irreversible loss of supportive structures. Its pathogenesis involves a complex interplay of microbial colonization, immune hyperactivation, and tissue‐destructive pathways.

Periodontitis initiates with the accumulation of dental plaque biofilm, a structured community of microorganisms [20]. Early colonizers (e.g., *Streptococcus sanguinis, Actinomyces*) adhere to the tooth surface, forming a glycoprotein pellicle. As the biofilm matures under anaerobic conditions, it transitions into a pathogenic consortium dominated by Gram‐negative anaerobes, including *Porphyromonas gingivalis, Tannerella forsythia*, and *Treponema denticola* (the “red complex”). These pathogens secrete virulence factors—such as gingipains produced by *Porphyromonas gingivalis* —that degrade host immunoglobulins, cytokines, and epithelial tight junctions, thereby impairing the integrity of mucosal barriers. The biofilm matrix protects bacteria from immune clearance and antimicrobial therapies, while quorum sensing coordinates the expression of virulence genes, enhancing pathogenicity.

The host response to pathogenic biofilms transitions from protective to destructive, driven by excessive inflammation and failed resolution. Innate immune receptors (TLR‐2/TLR‐4) recognize microbial components like LPS and peptidoglycan, activating the NF‐κB pathway and inducing pro‐inflammatory cytokines (IL‐1β, TNF‐α, IL‐6) that recruit neutrophils and macrophages to the periodontal pocket [21]. While neutrophils attempt to clear bacteria via myeloperoxidase and elastase, these enzymes also degrade collagen in the periodontal ligament, causing collateral tissue damage [22]. Concurrently, regulatory T cells (Tregs) are suppressed: *Porphyromonas gingivalis* degrades the Treg transcription factor Foxp3, reducing anti‐inflammatory signals (IL‐10, TGF‐β) [23]. This immune imbalance—marked by unchecked pro‐inflammatory responses and insufficient resolution—fuels persistent tissue destruction, despite ineffective antibody‐mediated pathogen clearance due to bacterial evasion mechanisms like intracellular persistence.

Periodontitis arises from a failure to resolve the conflict between pathogenic biofilms and dysregulated host immunity, leading to uncontrolled inflammation and tissue destruction. These factors create a self‐perpetuating cycle: pathogens induce inflammation, which damages tissues and creates a niche for further microbial invasion, while host aging and comorbidities erode protective responses. Additionally, systemic conditions like diabetes exacerbate periodontitis via advanced glycation end products (AGEs) activating RAGE receptors [24], while smoking inhibits neutrophil function and collagen synthesis [25]. This suggests a strong mechanistic linkage between periodontitis development and systemic diseases.

## 4 Epidemiological and Clinical Evidence of the Bidirectional Relationship

Epidemiological studies have established a significant dose‐dependent association between periodontitis and AD, with mounting evidence from longitudinal cohorts, case–control studies, and meta‐analyses [26, 27, 28, 29]. Individuals with periodontitis exhibit an increased risk of developing AD compared to healthy counterparts [30, 31, 32, 33]. A retrospective cohort study of 262,349 participants found chronic periodontitis (CP) was associated with a 5% higher risk of AD (aHR = 1.05, 95% CI = 1.00–1.11), highlighting CP as a potential modifiable risk factor for dementia independent of lifestyle behaviors [34]. The conclusion was also confirmed by a prospective cohort study of 90 ad patients, with periodontitis patients exhibiting accelerated cognitive decline [35]. Additionally, a quasi‐experimental study involving 177 periodontally treated patients and 409 untreated subjects found that periodontal treatment was associated with a favorable effect on AD‐related brain atrophy (adjusted effect: –0.41, 95% CI: −0.70 to −0.12), suggesting a potential role in mitigating preclinical AD [36]. These findings collectively highlight periodontitis as a modifiable risk factor with graded effects on AD susceptibility. However, a few inconsistent results have also been reported [36]. It has been hypothesized that these discrepancies are likely attributable to confounding variables, including variability in participant age, follow‐up duration, and baseline oral hygiene status. Prospective, standardized cohort studies with rigorous control for such confounders and uniform outcome measures are therefore warranted to resolve these inconsistencies and validate the proposed links.

Current research on the relationship between AD and periodontitis has primarily centered on the role of periodontitis in promoting the onset and progression of AD [6]. However, cross‐sectional data show that AD patients have higher bleeding on probing (BOP%) and greater plaque index (PLI) than age‐matched controls, reflecting a bidirectional association, whereby AD may also accelerate periodontal disease progression [37]. While the prevailing hypothesis attributes severe periodontal tissue destruction in AD patients to deteriorated oral hygiene maintenance due to cognitive dysfunction, recent clinical observations highlight potential synergistic interactions between neurodegeneration and periodontal inflammation [28]. These findings establish AD as a significant modifier of periodontitis progression, with epidemiological evidence linking cognitive impairment to accelerated tissue destruction [38]. Similarly, further epidemiological studies are still needed in the future.

## 5 Mechanistic Basis of the Bidirectional Association

### 5.1 Periodontitis to AD: Pathogen‐Mediated Neuroinflammation and Neuronal Damage

Periodontitis, a chronic bacterial inflammatory disease, involves periodontal pathogens whose pathogenic impacts extend well beyond localized tissue destruction. These microorganisms can translocate systemically via hematogenous, lymphatic, or neural pathways, enabling them to influence distant organ systems and contribute to the pathogenesis of extraporal diseases—processes collectively termed microbial translocation. Growing evidence indicates that *Porphyromonas gingivalis* and *Treponema denticola* —key pathogens of the periodontal “red complex”—can translocate to the brain, penetrating the BBB to induce neuroinflammation, synaptic dysfunction, and pathological cascades that contribute to cognitive impairment and AD [39, 40].

**FIGURE 2:**
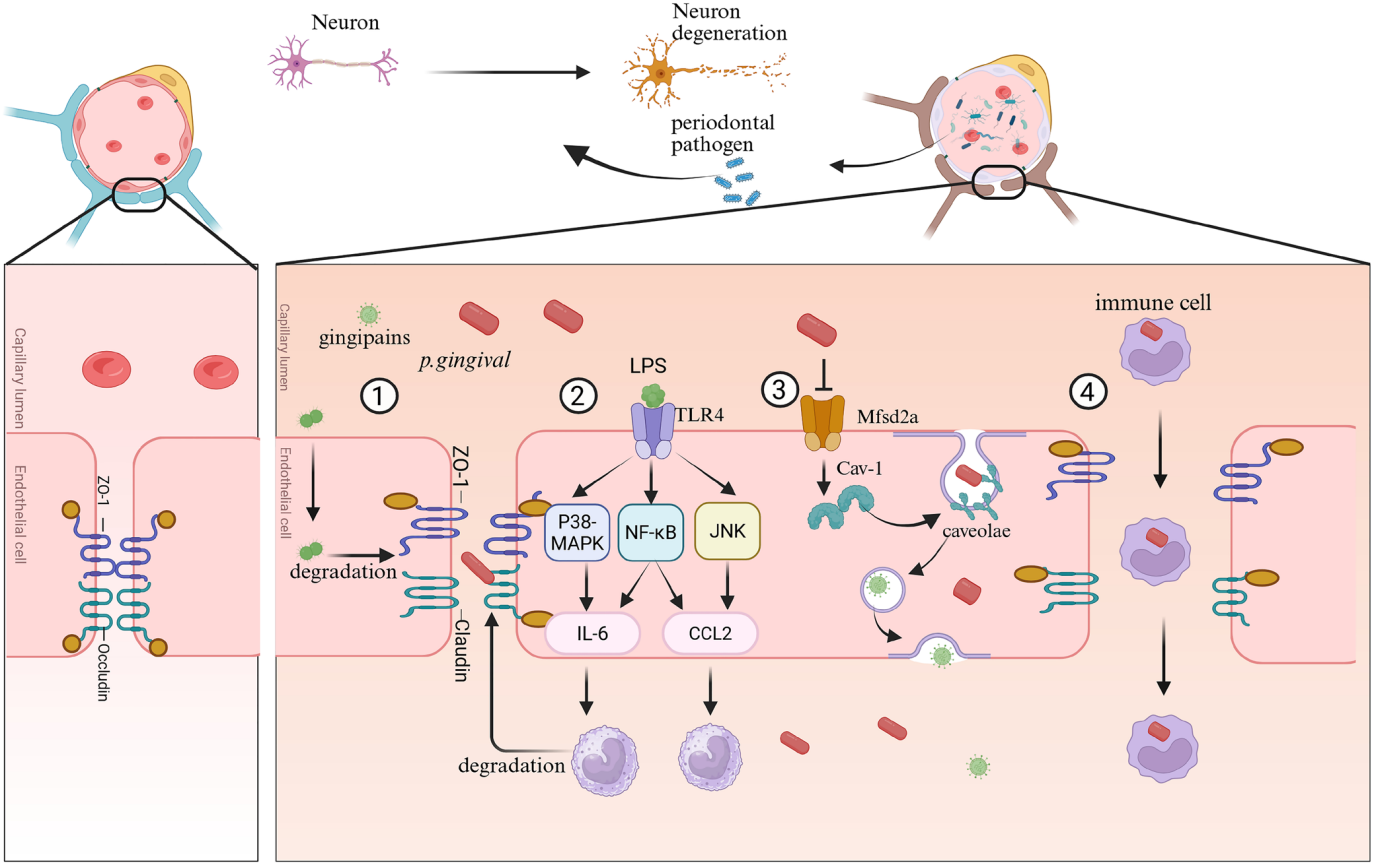
Multiple pathways in which periodontal pathogenic bacteria invade the blood–brain barrier. (1) Virulence factors secreted by *P. gingivalis* are able to increase BBB permeability by directly degrading tight junction proteins (e.g., ZO‐1 and occludin). (2) The virulence factors, such as LPS, can induce brain microvascular endothelial cells to secrete pro‐inflammatory chemokines via the P38‐MAPK, NF‐κB, and JNK signaling pathways. This process promotes the recruitment and accumulation of inflammatory cells while compromising the integrity of the blood–brain barrier by disrupting the tight junctions of endothelial cells. (3) *P. gingivalis* directly facilitates the invasion of pathogens into the brain by enhancing major facilitator superfamily domain containing 2a/Caveolin‐1 (Mfsd2a/Cav‐1)‐mediated transcytosis in brain microvascular endothelial cells. (4) Periodontal pathogens also induce immune cells to transmigrate across the blood–brain barrier.

Multiple routes are exploited by periodontal pathogens to invade the brain by crossing BBB. One primary route is hematogenous spread, whereby periodontal pathogens gain access to the circulatory system via compromised gingival capillaries, especially during episodes of bleeding or biofilm perturbation. Once in circulation, certain periodontal pathogens are capable of directly breaching the BBB to invade the brain parenchyma [9, 41]. BBB, composed of brain microvascular endothelial cells (BMECs) with tight junctions and supported by astrocytic end‐feet, pericytes, and a basement membrane, serves as a critical protective interface between the circulatory system and central nervous system (CNS). Recent evidence demonstrates that *Porphyromonas gingivalis* enables direct promotion of the invasion of pathogens into the brain by increasing major facilitator superfamily domain containing 2a/Caveolin‐1 (Mfsd2a/Cav‐1)‐mediated transcytosis in BMECs [42]. Additionally, *Porphyromonas gingivalis* exploits virulence factors like lipopolysaccharides (LPS) to increase BBB permeability and allow bacterial extravasation into the brain by directly degrading tight junction proteins (e.g., ZO‐1 and occluding) [43]. The virulence factors can also enter the cerebral parenchyma and initiate the inflammatory response to compromise BBB integrity [44]. In addition to direct invasion, periodontal pathogens also coerce immune cells to cross the blood–brain barrier; extracellular vesicle (EV) membrane fusion plays a critical role in cargo delivery, enabling the transfer of microbial virulence factors [45]. Besides compromising BBB integrity, pathogens, shielded within EVs, can directly traverse the BBB via vesicle‐mediated immune evasion mechanisms, triggering the accumulation of Aβ, a hallmark of AD pathogenesis [46]. These results demonstrate that hematogenous spread is the primary route through which pathogens invade the brain. Beyond hematogenous mechanisms, trigeminal nerve retrograde transport serves as an alternative route. Xiaoyang Ma et al. found fluorescein‐labeled *Porphyromonas gingivalis* extracellular vesicles in trigeminal ganglia and hippocampus following gingival exposure, indicating the trans‐nerve migration potential of pathogens to the brain [47]. Recent investigations underscore the intestinal pathway as a critical route for periodontal pathogens to influence AD progression. A seminal study by a Nanjing University research group demonstrated that oral microbiota derived from periodontitis patients, when administered via gavage to healthy mice, induced significant dysregulation of intestinal microbial homeostasis and perturbed intestinal immune balance [48]. These findings establish the oral‐intestinal axis as a novel transmission mechanism, operating independently of the bloodstream, by which periodontal pathogens may modulate neuroinflammatory and neurodegenerative processes associated with AD pathogenesis (Figure 2).

Upon invasion of the brain, periodontal pathogens initiate a dual‐pronged pathogenic process—neuroinflammation and direct neuronal damage—that collectively drive cognitive impairment. Neuroinflammation is primarily triggered by the activation of innate immune cells in the CNS through pathogen‐associated molecular patterns (PAMPs) [49]. Virulence factors such as LPS and gingipains from *Porphyromonas gingivalis* bind directly to Toll‐like receptor 4 (TLR4) and protease‐activated receptors (PARs) on microglia, activating the nuclear factor kappa–light–chain–enhancer of activated B cells (NF–κB) and mitogen‐activated protein kinase (MAPK) signaling pathways [50]. This activation elicits a persistent release of pro‐inflammatory cytokines, including IL‐1β and TNF‐α [50]. Subsequently, astrocytes are secondarily activated within the inflammatory microenvironment; they secrete chemokines (e.g., CXCL10), exacerbating immune cell infiltration and establishing an inflammatory cascade [51]. Chronic neuroinflammation disrupts synaptic plasticity and neurotransmitter transmission, and it further upregulates inducible nitric oxide synthase (iNOS), generating excessive nitric oxide (NO). The resultant oxidative stress and additional BBB damage create a permissive environment for pathogen dissemination and neurodegeneration. As a core pathogen in chronic periodontitis, *Tannerella forsythia* also plays a critical role in the progression of AD [52]. The key surface virulence molecules of this bacterium include BspA protein (a leucine‐rich repeat adhesion protein) and capsular polysaccharides [53]. Among them, the BspA protein can activate the inflammatory response of microglia and astrocytes by binding to TLR2 on the surface of host cells [54, 55]. Notably, accumulating evidence indicates that *Tannerella forsythia* acts synergistically with *Porphyromonas gingivalis* in pathological processes. In particular, cell extracts from *Tannerella forsythia* can stimulate the growth of *Porphyromonas gingivalis* [ 56]. Considering that *Porphyromonas gingivalis* has been implicated in promoting the progression of AD, it is plausible that *Porphyromonas gingivalis* and *Tannerella forsythia* exert a synergistic effect, which may in turn exacerbate AD‐associated pathogenesis. Moreover, it has been documented that both *Fusobacterium nucleatum* and *Aggregatibacter actinomycetemcomitans* cooperate synergistically with *Porphyromonas gingivalis* to drive the progression of neuroinflammation. The three periodontal pathogenic bacterial species exhibit a prominent metabolic complementarity, which serves as a critical mechanistic basis for their synergistic pathogenicity. Specifically, *Porphyromonas gingivalis* is auxotrophic for exogenous heme and vitamin B_12_ for proliferation [57]. In contrast, *Fusobacterium nucleatum* can efficiently sequester heme from the oral microenvironment via the heme‐binding protein (HbpA) localized on its outer membrane, while *Aggregatibacter actinomycetemcomitans* possesses the biosynthetic machinery to endogenously produce vitamin B_12_ [58, 59]. Through a well‐orchestrated “metabolic sharing” mechanism, *Fusobacterium nucleatum* and *Aggregatibacter actinomycetemcomitans* respectively supply *Porphyromonas gingivalis* with the indispensable heme and vitamin B_12_, thereby directly fulfilling *Porphyromonas gingivalis'*s nutritional requirements. This interspecies metabolic collaboration not only facilitates the robust proliferation of *Porphyromonas gingivalis* but also upregulates the transcriptional expression of *Porphyromonas gingivalis'*s virulence‐associated genes—including kgp and rgp, which encode the cysteine proteases gingipains (lysine‐specific gingipain and arginine‐specific gingipain, respectively)—leading to a marked enhancement of *Porphyromonas gingivalis* 's pathogenic potential. Upon infiltration into the CNS, *Fusobacterium nucleatum* , *Aggregatibacter actinomycetemcomitans* , and *Porphyromonas gingivalis* further synergize to trigger robust inflammatory responses. These pathogens release a repertoire of virulence factors, such as lipopolysaccharides, fimbriae, and toxins, which act in concert to activate two major CNS‐resident cell populations: microglia and astrocytes [60]. This coordinated activation elicits multi‐dimensional inflammatory signaling cascades, including the sustained activation of the TLR‐NF‐κB pathway, the assembly and activation of the NOD‐like receptor family pyrin domain‐containing 3 (NLRP3) inflammasome, and the initiation of the pyroptotic pathway. These inflammatory signaling events exhibit a mutually reinforcing and progressive amplification effect, ultimately culminating in a prominent “inflammatory amplification loop.” This loop not only exacerbates the local inflammatory milieu within the CNS but also sustains the progression of neuroinflammation, thereby contributing to the pathogenesis of neuroinflammatory and neurodegenerative disorders (Table 1).

**TABLE 1:** Novel research findings on the correlation between periodontitis and Alzheimer's disease.

Author	Main results	Methods of use	Research significance
Ciccotosto GD. et al. (2024) [40]	Chronic oral inoculation of female mice with *Porphyromonas gingivalis* alone induced all seven examined AD‐related brain pathologies, *Treponema denticola* alone caused specific ones, and their coinoculation led to limited pathologies with lower pathogenicity.	Animals: Forty 12‐week‐old female C57BL/6 mice, randomly assigned to 4 equal groups Intervention: Gingival margin inoculation at maxillary molars with *Porphyromonas gingivalis* or *Treponema denticola* Observation: IHC of Brain Tissue	The host brain response elicited by oral coinoculation was less than that elicited by each bacterium, suggesting coinoculation was less pathogenic
Lei S. et al. (2023) [42]	*Porphyromonas gingivalis* increases the permeability of blood–brain barrier endothelial cells via Mfsd2a/Cav‐1‐mediated transcytosis by the binding of gingipains to caveolin‐1 and the inhibition of major facilitator superfamily domain containing 2a expression.	Animals: 8‐week‐old healthy SD rats were randomly divided into three groups: high‐intensity group; low‐intensity group and control group Intervention: injected intravenously with *P. gingivalis* or PBS Observation: Transmission electron microscopy and Immunohistochemical observation	Mfsd2a/Cav‐1 mediated transcytosis is a key pathway governing BBB BMECs permeability induced by *P. gingivalis* , which may contribute to *P. gingivalis* /virulence factors entrance and the subsequent neurological impairments
Nonaka S. et al. (2022) [43]	Gingipains are delivered into human cerebral microvascular endothelial cells (hCMEC/D3) likely via outer membrane vesicles (OMVs), and directly degrade tight junction proteins Zonula occludens‐1 (ZO‐1) and occludin (both intracellularly and in vitro) to increase the permeability of the hCMEC/D3 cell monolayer.	Infection of hCMEC/D3 cells with WT *P. gingivalis*	Providing insight into the mechanism by which *P. gingivalis* crosses the blood–brain barrier (BBB) and potentially contributes to Alzheimer's disease (AD)‐related cognitive decline
Elashiry M. et al. (2024) [45]	Extracellular vesicles derived from the gingiva of mice and humans with periodontitis induced by *Porphyromonas gingivalis* contain *Pg* antigens (e.g., RGP, Mfa‐1) and proinflammatory cytokines (IL‐1β, IL‐6), can penetrate the blood–brain barrier (BBB) both in vitro (human 3D BBB model) and in vivo (uninfected recipient mice, colocalizing with hippocampal microglia), and promote BBB permeability.	Periodontitis model: C57B6 mice + *Porphyromonas gingivalis* oral gavage (6 weeks, control included) EXO prep/characterization: Mouse (gingiva/brain) + human (PD/healthy gingiva) EXOs; NTA/Western blot/TEM In vivo BBB penetration: Labeled mouse PD/Con EXOs → uninfected WT mice; IVIS/confocal tracking In vitro BBB assays: Human 3D model; TEER/FITC‐dextran for PD EXO‐induced integrity/permeability Component detection: Pg antigens (RGP/Mfa‐1) + cytokines (IL‐1β/IL‐6) in EXOs	Representing the first demonstration that oral microbial‐induced EXO may contribute to Alzheimer's disease (AD) pathogenesis by crossing the BBB
Ma X. et al. (2023) [47]	Gingival exposure to *Porphyromonas gingivalis* or its extracellular vesicles induces periodontitis, memory impairment‐like behaviors, hippocampal inflammation, reduced neuroprotective/BBB‐related protein expression, and gut dysbiosis with colitis, likely via pEV translocation to the brain through the trigeminal nerve and LPS entry via the periodontal blood pathway.	Cognitive behaviors were measured in the Y‐maze and novel object recognition tasks. Biomarkers were measured using ELISA, qPCR, immunofluorescence assay, and pyrosequencing	PG‐derived extracellular vesiclesmay be a remarkable risk factor for dementia
Lu J. et al. (2022) [48]	Gavage of periodontitis‐related salivary microbiota for two months impairs cognitive function, increases β‐amyloid accumulation and neuroinflammation, and induces gut microbial dysbiosis, intestinal pro‐inflammatory responses, intestinal barrier impairment, and systemic inflammation.	Sample collection: Isolate salivary microbiota from periodontitis patients and healthy individuals Animal intervention: Gavage periodontitis‐related salivary microbiota to APPswe/PS1ΔE9 (PAP) transgenic mice for 2 months Detection: Assess cognitive function, cerebral β‐amyloid accumulation, neuroinflammation, gut microbial dysbiosis, intestinal pro‐inflammation, intestinal barrier impairment, and systemic inflammation	Periodontitis may exacerbate AD pathogenesis through the gut‐brain axis via swallowed salivary microbiota, providing a novel perspective on AD etiology and intervention
Gong T. et al. (2022) [61]	Oral gavage of *Porphyromonas gingivalis* outer membrane vesicles (Pg OMVs, 4 mg/kg) for 8 weeks impairs memory/learning in 14‐month‐old mice, accumulates in hippocampus/cortex, reduces BBB tight junction proteins (ZO‐1, occludin, claudin‐5), activates astrocytes/microglia, and induces IL‐1β, tau Thr231 phosphorylation, and NLRP3 inflammasome activation (inhibited by MCC950 in vitro), with microglia‐conditioned media enhancing N2a neuron tau phosphorylation (attenuated by MCC950).	Animal: 14‐month‐old mice → oral gavage of Pg OMVs (4 mg/kg) or saline (q.o.d., 8 weeks) Behavioral tests: Open field, Morris water maze, Y‐maze In vivo: BBB permeability, hippocampal tight junction proteins (ZO‐1/occludin/claudin‐5), neuroinflammation, tau Thr231 phosphorylation, NLRP3 inflammasome; Pg OMVs localization (hippocampus/cortex) In vitro: BV2 + Pg OMVs (5 μg/mL) ± MCC950 (NLRP3 detection); N2a + microglia‐conditioned media ± MCC950 (tau phosphorylation assessment)	Pg OMVs trigger AD‐like pathologies via NLRP3 inflammasome
Jiang M. et al. (2021) [62]	Chronic systemic exposure to *Porphyromonas gingivalis* lipopolysaccharide (PgLPS) activates glycogen synthase kinase (GSK)‐3β in microglia and neurons, induces microglial tumor necrosis factor (TNF)‐α production, thereby triggering neuronal tau hyperphosphorylation, neuroinflammation, and learning and memory deficits in APPNL‐F/NL‐F mice without altering amyloid (A)β1‐42 expression	In Vivo Model: 10‐month‐old AD model Intervention: PgLPS (1 mg/kg, i.p., daily × 3 weeks) Behavioral Test: Passive avoidance test (learning/memory) Techniques: IHC, Western blotting, ELISA 2 In Vitro Cell Models: MG6 microglia, N2a neurons Assays: TNF‐α: qPCR (mRNA), Western blotting (protein)	Inhibiting GSK3β activation may help delay the periodontitis‐promoted pathological progression of Alzheimer's disease
Wu B. et al. (2024) [63]	AD patients exhibit an increased osteoclastogenesis signature in blood (positively correlated with impaired peripheral Aβ clearance by immune cells), while long‐term pharmacological blockade of osteoclasts with Alendronate in APP23 transgenic AD mice improves peripheral monocyte Aβ‐degrading enzyme expression, reduces Aβ deposition, and mitigates memory decline	Targeted analysis of public whole blood transcriptomes from AD patients to identify molecular signatures and pathways linked to OC hyperactivation Using APP23 TG AD mice, long‐term Alendronate‐mediated OC inhibition to evaluate effects on AD pathology (Aβ deposition, Aβ‐degrading enzyme expression) and memory function	Osteoclasts promote AD development/progression possibly via modulating peripheral immunity and highlighting the potential of osteoporosis prevention in alleviating cognitive burden
Weng Y. et al. (2020) [64]	Triggering Receptor Expressed on Myeloid Cells 2 (Trem2) is significantly upregulated in the alveolar bones of patients with chronic periodontitis, and it amplifies reactive oxygen species (ROS) signals in osteoclasts through a Trem2/DAP12/Syk‐dependent signaling cascade; furthermore, soluble Aβ42 oligomers (Aβo) in the periodontitis microenvironment can directly bind to Trem2 to enhance this signal and osteoclastogenesis, while conditional knockout of Trem2 in osteoclasts inhibits alveolar bone resorption in mice with periodontitis	Clinical sample analysis: RNA‐seq was used to detect Trem2 expression in alveolar bones of chronic periodontitis patients Animal model study: A periodontitis mouse model was established; Trem2 was conditionally knocked out in osteoclasts, and alveolar bone resorption was compared between knockout and control groups Molecular mechanism exploration: Molecular biology techniques verified the Trem2/DAP12/Syk cascade in osteoclast ROS signal amplification, and explored the interaction between Aβo and Trem2 and its regulatory effect on osteoclastogenesis	Trem2 is a potential target for the prevention and treatment of bone destruction in periodontitis and AD
Catalina Arévalo‐Caro et al. (2025) [65]	This study investigates the potential link between periodontal disease (PD) and Alzheimer's disease (AD) through the apolipoprotein E gene ε4 allele (APOE4)—a primary genetic risk factor for AD associated with chronic inflammatory conditions like PD, which potentiates AD development and progression	The Joanna Briggs Institute methodology and PRISMA guidelines were followed. The search included articles published in PubMed and Embase, focusing on human studies, and excluding case series, in vitro studies, reviews, and animal studies	APOE4 may link PD and AD through shared genetic variants, inflammatory pathways, and dyslipidemia

Direct neuronal damage by periodontal pathogens relies on their specialized virulence mechanisms [46]. Gingipains (RgpA and Kgp) secreted by *Porphyromonas gingivalis* , as cysteine proteases, selectively cleave neuronal surface receptors, extracellular matrix proteins, and key signaling molecules. Studies have demonstrated that gingipains degrade neural cell adhesion molecule (NCAM) and nerve growth factor (NGF) receptor TrkA, thereby disrupting inter‐neuronal connections and inhibiting axonal growth [47]. Additionally, gingipains process the β‐amyloid precursor protein into neurotoxic Aβ fragments and promote abnormal phosphorylation of tau protein, accelerating the formation of neurofibrillary tangles [66]. Anaerobic pathogens like *Treponema denticola* directly damage neuronal membranes through outer‐membrane proteins and hemolysins, inducing mitochondrial dysfunction and apoptosis. Recent research has revealed that EVs of periodontal pathogens can traverse the BBB, carrying virulence factors [45]. Through membrane fusion, EVs deliver gingipains and LPS directly into the neuronal cytoplasm, triggering caspase‐3‐dependent apoptosis and endoplasmic reticulum stress [61]. In addition, certain periodontal pathogenic bacteria can secrete specific virulence factors that directly target kinases associated with tau protein phosphorylation in neurons, triggering their abnormal activation and thereby accelerating the process of tau protein phosphorylation. Among these bacteria, *Porphyromonas gingivalis* has the most well‐defined role and the most sufficient research evidence [61]. The gingipains secreted by this bacterium not only cleave the APP to promote the production of Aβ, but also directly act on the key regulatory kinases of tau protein phosphorylation, namely p38 mitogen‐activated protein kinase and glycogen synthase kinase‐3β (GSK‐3β) [62]. Researchers found that *Porphyromonas gingivalis* could significantly upregulate the phosphorylation level of p38 MAPK [67]. The activated p38 MAPK could directly phosphorylate specific sites of the tau protein, which are exactly the main phosphorylation sites of the tau protein in NFTs in the brains of AD patients. And other periodontal pathogenic bacteria can also promote the process of tau protein hyperphosphorylation. *Tannerella forsythia* activates the MAPK inflammatory pathway by binding to immune cells' TLR2 through the BspA protein, thereby promoting the release of pro‐inflammatory factors. *Fusobacterium nucleatum* enhances the permeability of the blood–brain barrier, allowing peripheral inflammatory cells to infiltrate and activate, indirectly promote tau protein hyperphosphorylation [68]. Moreover, chronic neuroinflammation leads to abnormal function of astrocytes, reduces the supply of PP2A, forming a “inflammation‐insufficient phosphatase‐tau accumulation” vicious cycle, accelerating the formation of NFTs and neuronal death.

While Aβ and tau pathology have been the focus of periodontitis‐AD research, the involvement of α‐synuclein—a presynaptic protein aggregated in Parkinson's disease (PD) and dementia with Lewy bodies (DLB)—remains largely unexplored. α‐synuclein aggregation is driven by neuroinflammation, oxidative stress, and microbial insults [69], all of which are hallmarks of periodontitis. However, direct evidence linking periodontal pathogens to α‐synuclein misfolding or propagation is lacking. Several open questions persist: (1) Do periodontal pathogens (e.g., *P. gingivalis* , *T. forsythia* ) or their virulence factors induce α‐synuclein phosphorylation or oligomerization in neurons or glia? (2) Can periodontitis‐associated neuroinflammation promote the prion‐like spread of α‐synuclein aggregates in the brain? (3) Is there an overlap between periodontitis‐induced tau/Aβ pathology and α‐synuclein aggregation, potentially exacerbating mixed dementia phenotypes? Preliminary in vitro data suggest that LPS from *P. gingivalis* may upregulate α‐synuclein expression in neuronal cells, but in vivo validation and mechanistic studies are absent. Given the comorbidity of AD and synucleinopathies in aging populations, clarifying the role of α‐synuclein in the oral‐brain axis is essential to fully understand the spectrum of periodontitis‐associated neurodegeneration.

In summary, these multi‐dimensional pathogenic pathways that extend from local infections to neurodegenerative diseases provide crucial theoretical basis for the development of prevention and treatment strategies for AD and other cognitive disorders.

### 5.2 AD to Periodontitis: Neurodegeneration‐Mediated Disruption of Bone Homeostasis

AD‐related neurodegeneration impairs oral health through autonomic and behavioral mechanisms, which is regarded as the primary cause of periodontal destruction in AD patients. However, several studies have unveiled the inherent link between AD and periodontitis, suggesting that beyond oral hygiene practices, AD itself influences the progression of periodontitis [70]. Alveolar bone loss represents a key clinical manifestation of periodontitis, with bone homeostasis imbalance serving as a critical pathological basis for bone mass loss. Existing literature demonstrates that AD patients exhibit disruptions in bone homeostasis, further corroborating the role of AD in driving periodontal disease progression [71].

**FIGURE 3:**
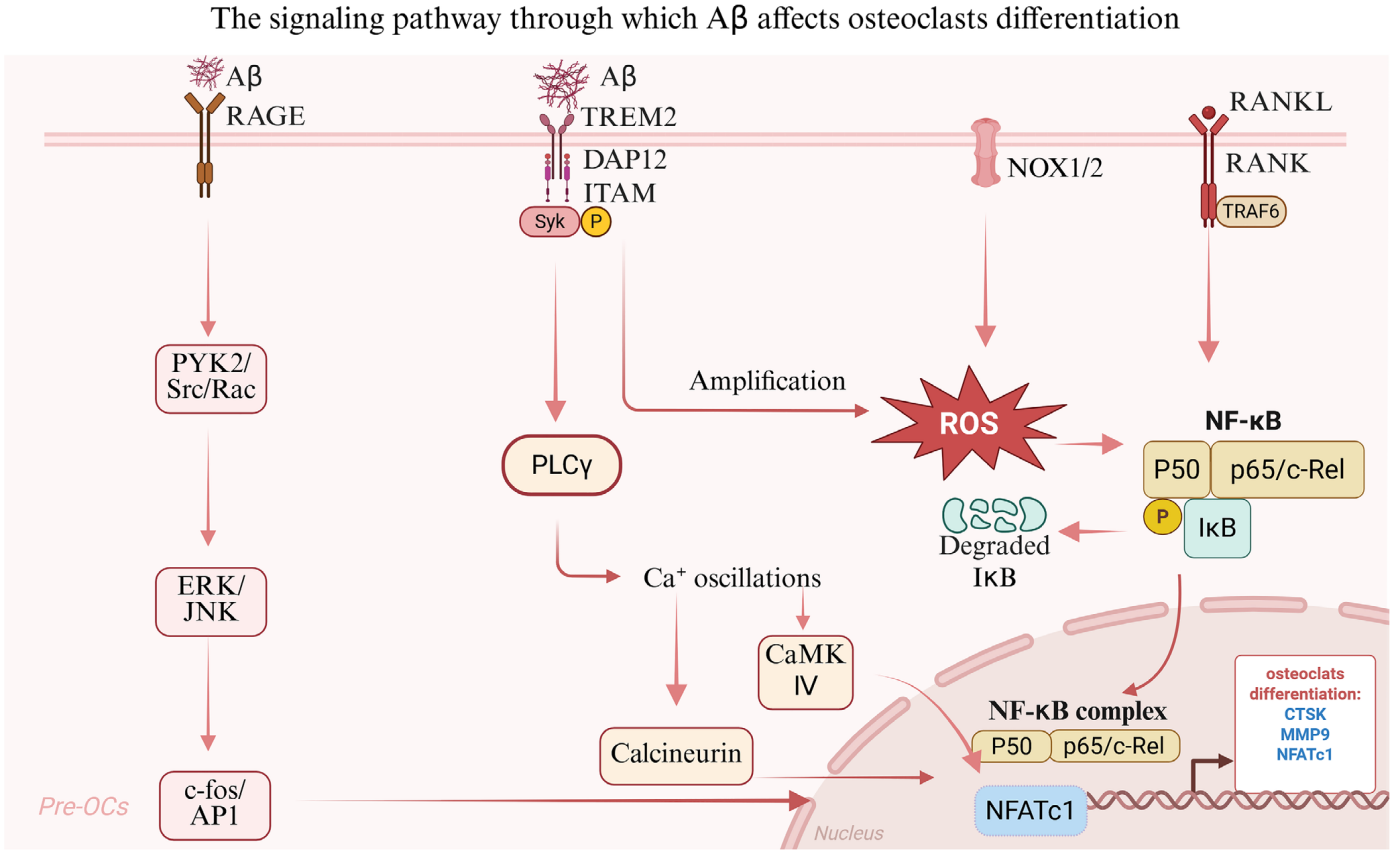
Multiple signaling pathways through which Aβ affects osteoclasts. Aβ drives osteoclast differentiation and activation via multiple mechanisms: (1) interaction with the Receptor for Advanced Glycation Endproducts (RAGE)—a key mediator of age‐related inflammation—promotes the expression of osteoclast differentiation genes. (2) binding to Triggering Receptor Expressed on Myeloid Cells 2 (TREM2) initiates a reactive oxygen species (ROS)‐dependent feedforward loop or calcium oscillation signaling cascades that accelerate osteoclast differentiation.

As a central pathologic hallmark of AD, Aβ in the brain can be excreted to peripheral organs and deposited in skeletal system, where it disrupts bone homeostasis—a process increasingly linked to aging‐related osteopenia. While direct evidence for Aβ exacerbating periodontitis progression remains inconclusive, a robust body of literature establishes its role in dysregulating bone remodeling and inducing bone mass loss, with mechanistic insights deeply rooted in aging‐associated cellular dysfunction. Aβ exerts age‐dependent effects on osteoclastogenesis, a key driver of bone resorption in aging populations [63]. Aβ promotes osteoclast differentiation and activation through multiple pathways: (1) engagement of the Receptor for Advanced Glycation Endproducts (RAGE), a receptor critically involved in age‐related inflammation, amplifies osteoclast‐mediated bone resorption [72]; (2) synergistic activation of NF‐κB, ERK, and calcium oscillation signaling enhances RANKL‐induced osteoclast activation, a pathway often dysregulated in senescent bone microenvironments [73, 74]; (3) interaction with Triggering Receptor Expressed on Myeloid Cells 2 (TREM2) initiates a reactive oxygen species (ROS)‐driven feedforward loop, accelerating osteoclast differentiation [64]; Notably, these pro‐resorptive effects are partially mitigated in aged individuals by upregulation of soluble RAGE (sRAGE) and osteoprotegerin (OPG), though this compensation coincides with impaired bone remodeling and increased fracture susceptibility—hallmarks of aging [72]. Concurrently, Aβ undermines osteoblast function and mesenchymal stem cell (MSC) fate commitment, critical processes for bone formation and renewal. Osteoblast‐specific APP overexpression in TgAPPswe‐OCN mice recapitulates trabecular bone loss [75], while young Tg2576 mice exhibit adipogenic skewing of MSCs, highlighting Aβ's dual role in suppressing osteogenesis and promoting marrow adiposity—phenotypes strongly associated with skeletal aging. Oxidative stress and mitochondrial dysfunction emerge as unifying mechanisms linking Aβ to skeletal dysfunction. Antioxidant interventions (e.g., N‐acetyl‐L‐cysteine) rescue osteogenic potential in Tg2576‐derived MSCs and alleviate osteopenia in vivo. Our recent work also demonstrates that Aβ‐induced mitochondrial fission impairs type H blood vessels—a key regulator of bone vascular osteogenesis—in APP/PS1 mice [76]. These findings converge on Aβ‐induced imbalance of bone homeostasis as a potential central node in periodontal destruction in AD patients (Figure 3).

As a critical component of the brain‐bone axis governing skeletal physiology, the autonomic nervous system (ANS) has emerged as a key regulator of bone metabolism. Notably, sympathetic nerve hyperactivity has been documented in AD patients [77], establishing a potential neuro‐skeletal connection. Preclinical studies have demonstrated that chemical sympathectomy alleviates inflammation‐induced alveolar bone loss [78], implicating excessive sympathetic activation as a modifiable target for bone preservation. Mechanistically, noradrenergic terminals within the bone microenvironment release norepinephrine (NE), which binds to β₂‐adrenergic receptors (β₂ARs) on osteoblasts and osteocytes. This signaling cascade exerts dual effects: (1) suppressing osteoblastic bone formation; and (2) upregulating receptor activator of nuclear factor kappa‐B ligand (RANKL) expression, thereby promoting osteoclastogenesis and enhancing bone resorption [79, 80]. Additionally, osteoclast progenitors themselves express β₂ARs; however, there is still some controversy over its direct role in sympathetic stimulation directly accelerates their differentiation into mature bone‐resorbing cells [81]. Complementing sympathetic function, the parasympathetic nervous system (PNS) maintains skeletal balance via counter‐regulatory mechanisms. While direct evidence linking PNS dysfunction to periodontitis in AD remains lacking, AD patients exhibit characteristic parasympathetic impairments—including gastrointestinal dysmotility and dysregulated blood pressure control [82]. These systemic manifestations suggest a broader role for PNS insufficiency in mediating inflammatory bone loss, potentially through disrupted neuro‐immune crosstalk in periodontal tissues.

Additionally, a multifaceted disruption of the endocrine system represents a critical pathway through which AD impacts bone remodeling, with core mechanisms embedded in the bidirectional interplay between neurodegeneration and dysregulated endocrine networks. These interactions fundamentally disrupt the dynamic equilibrium between bone formation and resorption, driving pathological skewed remodeling. Hyperactivation of the hypothalamic–pituitary–adrenal (HPA) axis in AD leads to sustained hypercortisolism [83], which directly inhibits osteoblast proliferation and differentiation while reducing bone matrix synthesis and enhances osteoclast precursor recruitment and activity to promote bone resorption [84, 85]. Cortisol also disrupts vitamin D metabolism by inhibiting renal proximal tubule 1α‐hydroxylase activity, decreasing intestinal calcium absorption and indirectly exacerbating bone loss [86]. Sex hormone imbalance plays a critical role in bone remodeling disorders: reduced estrogen levels in postmenopausal female AD patients weaken its inhibitory effect on osteoclasts (by decreasing secretion of osteoprotegerin, an inhibitor of receptor activator of nuclear factor κB ligand [RANKL]) and reduce osteoblastic sensitivity to growth factors [87], while testosterone deficiency in male AD patients impairs bone formation by downregulating androgen receptor expression in osteoblasts and inhibiting IGF‐1 synthesis, potentially synergizing with cortisol to exacerbate muscle loss and reduce skeletal mechanical load stimulation. As a hallmark of AD, glycometabolic dysfunction (“type 3 diabetes”) impairs osteoblastic insulin receptor signaling via insulin resistance, inhibiting glucose transport into osteoblasts and collagen synthesis, while reduced glucagon‐like peptide‐1 (GLP‐1) secretion not only exacerbates glycemic fluctuations but also indirectly promotes bone resorption by weakening inhibition of osteoclasts [88]. Hypothalamic–pituitary‐growth hormone axis dysfunction, leading to deficiencies in growth hormone (GH) and insulin‐like growth factor‐1 (IGF‐1), directly impairs osteoblast proliferation and differentiation; IGF‐1 insufficiency further reduces type I collagen synthesis and inhibits osteoclast apoptosis, disrupting bone remodeling bidirectionally [88]. Thyroid dysfunction (e.g., low T3 syndrome) slows bone turnover and affects mineralization by downregulating thyroid hormone receptor β expression in osteoblasts [89]. Additionally, circadian rhythm disruption of melatonin secretion in AD may increase bone marrow adipocyte differentiation (marrow adiposity inhibits osteogenesis) via the suprachiasmatic nucleus‐sympathetic nerve pathway, while melatonin deficiency may weaken its inhibitory effect on osteoclast activity [88]. These endocrine abnormalities collectively act through multiple synergistic pathways, ultimately leading to osteoclast‐dominated bone resorption exceeding osteoblast‐mediated bone formation, inducing osteoporosis, bone microarchitecture deterioration, and increased fracture risk, thus creating a vicious cycle between AD and bone metabolic disorders.

Although the specific mechanisms described above remain to be validated in periodontitis, the endocrine‐mediated pathways through which AD influences bone metabolism may underlie the accelerated progression of periodontitis in individuals with preclinical AD. Prospective translational studies are therefore warranted to investigate this hypothesis and clarify the bidirectional relationship between neurodegenerative processes and periodontal bone loss.

### 5.3 Age‐Related Factors Amplifying the Bidirectional Relationship

Periodontitis and AD both fall under the category of age‐related diseases and aging primes a bidirectional exacerbation between periodontitis and AD through two interlinked processes: immunosenescence and the chronic low‐grade systemic inflammation characteristic of aging. These aging‐related shared mechanisms converge to impair clearance of oral pathogens and brain protein aggregates while fostering a pro‐inflammatory microenvironment, creating a vicious cycle that amplifies disease progression in both systems.

With age, naïve T cell pools shrink, while memory T cells accumulate in a senescent state, exhibiting reduced proliferation and skewed cytokine profiles [90]. In periodontitis, this compromises Th1/Th17‐mediated defense against *Porphyromonas gingivalis* , leading to impaired macrophage activation and 25% reduced phagocytosis of *Porphyromonas gingivalis* in aged gingival tissues [91]. Concurrently, in AD, senescent T cells fail to regulate microglial pro‐inflammatory responses, while B cells produce low‐affinity antibodies against Aβ, decreasing humoral clearance of Aβ oligomers [92]. Autopsies reveal reduced Aβ antibody titers in AD patients with periodontitis, correlating with higher plaque burden.

Aging also impairs neutrophil chemotaxis and phagocytic efficiency, delaying migration to periodontal pockets and reducing bacterial killing. This allows *Porphyromonas gingivalis* to persist and secrete virulence factors (e.g., gingipains), accelerating tissue destruction [93]. In the brain, aged neutrophils and monocytes exhibit defective clearance of Aβ aggregates: peripheral blood monocytes from AD patients show reduced phagocytosis of Aβ_42_ and increased release of IL‐1β, fostering neuroinflammation. Neutrophil extracellular traps (NETs) in aged individuals are hyperactivated but dysfunctional, releasing DNA citrullination products that cross‐react with tau, promoting tangle formation [94].

Brain‐resident microglia undergo senescence in aging, adopting a dystrophic phenotype with reduced phagocytic capacity and heightened secretion of SASP factors. This “primed” state amplifies neuroinflammation, while dendritic cells (DCs) in both oral and brain tissues exhibit reduced MHC‐II expression and increased IL‐10 secretion, promoting immune tolerance to pathogens and protein aggregates [95]. In the periodontium, senescent DCs fail to activate protective T cell responses, allowing biofilm persistence; in the brain, they exacerbate Aβ‐induced microglial activation, forming a feedback loop of dysfunctional immunity [96, 97].

Genetic factors are innate determinants of the pathogenesis of AD and periodontitis [98]. Certain genetic variants increase the susceptibility to both diseases by regulating shared pathways such as immune response, inflammatory reaction, and metabolic function, serving as a “genetic link” connecting the two. The apolipoprotein E (APOE) gene is currently recognized as a common high‐risk gene for AD and periodontitis, and its ε4 allele exerts bidirectional disease‐promoting effects through multiple mechanisms [65]. APOEε4 accelerates the progression of AD through three pathways: first, it reduces Aβ clearance efficiency (APOEε4 has weak binding ability to Aβ and cannot effectively mediate the transport of Aβ across the BBB or its phagocytosis by microglia); second, it promotes Aβ aggregation (APOEε4 can induce Aβ to form more toxic oligomers); third, it exacerbates neuroinflammation (APOEε4 can activate microglia to secrete more pro‐inflammatory factors) [99]. APOEε4 is also considered to be closely related to the progression of periodontal disease [100]. However, at present, there is a lack of corresponding basic research to verify this conclusion. We speculate that APOEε4 may influence the progression of periodontitis through the following aspects. First, it impairs the phagocytic capacity of neutrophils and macrophages against periodontal pathogens (APOEε4 affects the lipid metabolism of immune cells, reducing their antibacterial activity) [101]; second, it exacerbates periodontal inflammation (APOEε4 can increase the expression of TNF‐α and IL‐1β in periodontal tissues, amplifying the inflammatory response) [102]; third, it may reduce the repair capacity of periodontal tissues. In addition, triggering receptor expressed on myeloid cells 2 (TREM2) is a key regulatory factor for microglia and macrophages [103, 104]. Its genetic mutations can impair the ability of microglia to clear Aβ, increasing the risk of AD; at the same time, this mutation also reduces the bactericidal effect of macrophages on periodontal pathogens, leading to increased susceptibility to periodontitis and more severe disease conditions.

Lifestyle is a core acquired influencing factor for the pathogenesis of AD and periodontitis. Unhealthy living habits such as smoking and sleep disorders drive the progression of both diseases by long‐term affecting systemic metabolism, immune status, and oxidative stress levels, and there is a synergistic effect among these factors, forming a “multifactorial superimposed” risk effect [105]. Smoking is the strongest modifiable risk factor and can cause cross‐system damage. Harmful substances in tobacco, such as nicotine, tar, and heavy metals, can cross the BBB and directly damage neuronal cell membranes and DNA; at the same time, smoking activates the systemic oxidative stress response, promoting the production of reactive oxygen species (ROS) in the brain and accelerating Aβ aggregation and abnormal tau phosphorylation; in addition, smoking also reduces cerebral blood flow (especially in cognitive‐critical brain regions such as the hippocampus), exacerbating cerebral ischemia and hypoxia and impairing synaptic plasticity. Furthermore, smoking inhibits the proliferation of gingival fibroblasts and collagen synthesis, impairing the repair capacity of periodontal tissues; tobacco smoke reduces the concentration of antibacterial components such as lysozyme and mucin in saliva, increasing the colonization of pathogens such as *Porphyromonas gingivalis* and *Tannerella forsythia* ; at the same time, smoking also reduces blood supply to periodontal tissues, lowering the chemotaxis and phagocytic efficiency of neutrophils, resulting in more severe periodontal inflammation and faster alveolar bone resorption [98]. In addition, sleep disorders (e.g., insomnia, obstructive sleep apnea) also act as bidirectional risk factors for AD and periodontitis by affecting the “clearance‐repair” process [106, 107].

Systemic chronic diseases such as diabetes, cardiovascular disease (CVD), and chronic obstructive pulmonary disease (COPD) accelerate the progression of both AD and periodontitis through shared pathways of “chronic inflammation‐immune dysregulation‐metabolic disorder,” forming a cross‐system pathological chain of “systemic disease → local disease (periodontitis) → brain disease (AD)” [108]. In terms of promoting AD, cerebral insulin resistance in type 2 diabetes mellitus (T2DM) inhibits the activity of Aβ‐clearing enzymes, advanced glycation end products (AGEs) produced by hyperglycemia damage neurons and the BBB, and cerebral microangiopathy reduces blood supply to cognitive‐critical brain regions [109]; cerebral microangiopathy (sclerosis, embolism) in CVD causes cerebral ischemia and hypoxia, promoting Aβ deposition and tau protein tangles, systemic inflammation (e.g., elevated C‐reactive protein, IL‐6) activates microglia, and hypertension also damages the BBB [110]; long‐term hypoxemia in COPD damages cognitive brain regions such as the hippocampus, pro‐inflammatory factors (TNF‐α, IL‐8) released from the lungs spread to the brain through an “inflammatory cascade,” and comorbid sleep apnea further exacerbates Aβ clearance disorders [111]. In terms of promoting periodontitis, all three diseases exert their effects by impairing the local defense and repair capacity of the periodontium, enhancing pathogen virulence, or exacerbating inflammatory responses. Hyperglycemia in T2DM provides “nutritional support” for periodontal pathogens (e.g., *Porphyromonas gingivalis* ) and also damages neutrophil phagocytic function and increases the level of inflammatory factors [21]; impaired vascular endothelial function in CVD reduces blood supply to periodontal tissues, coagulation disorders (e.g., platelet activation) increase the risk of periodontal bleeding, and anticoagulant drugs (e.g., aspirin) taken by patients may also affect the effect of periodontal treatment; coughing and expectoration in COPD patients increase the colonization of oral pathogens (e.g., *Klebsiella pneumoniae* , *Pseudomonas aeruginosa* ), long‐term use of glucocorticoids inhibits immune function [112], and hypoxemia also reduces blood supply to the periodontium, promoting the progression of periodontitis.

## 6 Therapeutic Implications and Interventional Strategies

Oral care for patients with AD must be adjusted according to the disease stages, with the core principle of adapting to changes in patients' cognitive and self‐care abilities, and reducing the risks of oral diseases and related complications (Table 2).

**TABLE 2:** Phased oral care strategies for patients with Alzheimer's disease.

Stage	Core objectives	Specific care measures	Key considerations	Reference citations
Early stage	Prevention + Development of Independent Care Habits	Implement fluoride therapy and shorten intervals between oral examinations and scaling Prioritize simple and durable restorative treatment options Provide adaptive cleaning tools and use memory aids such as color coding or the 5S methodology Conduct health education for patients and caregivers Promptly provide fixed/removable dental prostheses for edentulism and offer guidance on prosthesis cleaning	Maintain patients' independent care capabilities and avoid over‐reliance on caregivers prematurely; reinforce memory through simple and understandable methods to ensure the sustainability of care measures	[113, 114, 115]
Moderate stage	Managing Resistant Behaviors + Maintaining Oral Health Status	Dental procedures: Use mouth props to prevent biting, adopt semi‐supine/supine positions to reduce aspiration risk, and shorten appointment durations Recommend Atraumatic Restorative Techniques (ART) for caries treatment Adopt the “tell‐show‐do” communication approach and have caregivers lead daily oral care Monitor xerostomia closely and use artificial saliva if necessary Regularly inspect dentures for looseness or damage	Focus on alleviating patients' resistant behaviors with gentle and efficient operations; strengthen caregivers' care capabilities to prevent complications such as aspiration and xerostomia	[116, 117]
Late stage	Palliative Care + Emergency Management	Adopt simplified cleaning methods Use topical antimicrobial gels for oral infections and oral analgesics for severe toothache Establish a multidisciplinary team to assess aspiration pneumonia risk; consider oral cleaning under general anesthesia for severe bacterial accumulation; Perform emergency interventions only for life‐threatening oral issues Train caregivers in infection sign recognition and basic first aid skills	Prioritize patient dignity with gentle care operations; address life‐threatening emergencies as the top priority; provide comprehensive support to caregivers to reduce complication risks	[118, 119]

In the early stage, the focus is on prevention and the establishment of independent care habits [113]. Since patients still have relatively intact cognitive function, fluoride therapy should be implemented to prevent dental caries, and the intervals between oral examinations and scaling should be shortened. Simple and durable restorations are preferred to avoid complex treatments in later stages. Adaptive tools such as electric toothbrushes and water flossers should be provided, and strategies like color marking or the 5S methodology (Sort, Set in order, Shine, Standardize, Sustain) can be used to assist with memory [114]. Meanwhile, both patients and caregivers should receive education on proper cleaning techniques and dietary control (e.g., reducing sugar intake). Additionally, attention should be paid to maintaining masticatory function—dental prostheses (fixed or removable) should be provided promptly for tooth loss, and guidance on prosthesis cleaning should be given [115].

In the moderate stage, the key lies in managing resistant behaviors and maintaining oral health status. Patients often experience significant cognitive decline and behavioral resistance (e.g., refusing tooth brushing or dental treatment) [116]. During dental procedures, mouth props should be used to prevent biting, semi‐supine or supine positions to reduce aspiration risk, and appointment durations should be shortened (e.g., 15–30 min per session). Atraumatic restorative techniques (ART) are recommended for caries treatment. The “tell‐show‐do” communication method (explaining, demonstrating, and performing) should be adopted to reduce resistance, and caregivers should take the lead in daily oral care (e.g., assisting with brushing). Xerostomia should be closely monitored, and artificial saliva should be used when necessary [117]. For patients with dentures, caregivers should check for looseness or damage regularly to prevent aspiration.

In the late stage, palliative care and emergency management are prioritized. Patients with severe cognitive impairment (e.g., inability to recognize relatives or perform independent activities) require simplified cleaning, such as wiping the oral mucosa with saline‐soaked cotton swabs. Local antimicrobial gels (e.g., metronidazole gel) can be used for oral infections, and oral analgesics for severe toothache [118]. A multidisciplinary team (including geriatrics and respiratory departments) should assess the risk of aspiration pneumonia; in cases of severe oral bacterial accumulation, oral cleaning under general anesthesia may be considered [119]. Only life‐threatening oral issues (e.g., severe maxillofacial infections) require emergency intervention. Caregivers should be educated on identifying infection signs and basic first aid, while ensuring gentle care to respect patients' dignity.

Overall, oral care for AD patients requires stage‐specific adaptation, with continuous guidance for caregivers to maintain oral health and improve patients' quality of life.

## 7 Challenges and Future Directions

Most studies exploring the periodontitis‐AD relationship are observational, establishing associations but not causal links. High‐quality longitudinal cohorts and randomized controlled trials (RCTs) are critical to disentangle causation, as current evidence cannot definitively prove that periodontal infection drives AD pathogenesis or vice versa. Additionally, pathogen‐specific mechanisms remain unclear: while *Porphyromonas gingivalis* is most studied, the distinct roles of other periodontal pathogens (e.g., *Tannerella forsythia, Fusobacterium nucleatum*) and their synergistic effects in AD pathology are underinvestigated. Heterogeneity in microbial composition and disease stages further complicates understanding of specific bacterial contributions, highlighting the need for mechanistic studies dissecting species‐specific impacts on neuroinflammation and amyloid/tau pathology.

Developing oral microbiota‐based biomarkers, such as salivary DNA of periodontal pathogens, offers non‐invasive potential for early AD screening. Advanced techniques like metagenomic sequencing and machine learning can identify pathogen‐host interaction patterns, with preclinical studies linking elevated salivary *Porphyromonas gingivalis* DNA to brain amyloid load. Concurrently, exploring dynamic oral‐brain axis mechanisms in aging—inclusive of microbial translocation, systemic inflammation, and barrier dysfunction—facilitates interdisciplinary interventions. Integrated strategies combining periodontal therapy (scaling, probiotics), cognitive training, and microbiota modulation aim to disrupt bidirectional inflammatory loops, with ongoing trials assessing their impact on cognitive decline and oral health outcomes in at‐risk populations.

## 8 Conclusion

The bidirectional relationship between AD and periodontitis is deeply rooted in shared pathological mechanisms, including microbial translocation, neuroinflammation, and disrupted bone homeostasis, with aging exacerbating this interplay via immunosenescence and inflammaging. Epidemiologically, periodontitis increases AD risk by 1.5–2‐fold, while AD patients exhibit a 35% higher periodontitis prevalence, forming a reciprocal inflammatory loop. Pathologically, oral pathogens drive Aβ/tau pathology via hematogenous/neural invasion, whereas AD impairs oral mucosal immunity and bone remodeling. Clinical evidence supports periodontal therapy as a modifiable strategy to reduce systemic inflammation, yet causal links and pathogen‐specific mechanisms remain unclear. Future research must prioritize longitudinal trials, mechanistic dissection of the oral‐brain axis, and interdisciplinary interventions integrating dental, neurological, and geriatric approaches to develop targeted preventive strategies for aging populations.

## Author Contributions

W.Z., H.L. and M.L. wrote and revised the manuscript. Y.C. and Y.L. were involved in collecting data. W.Z. and S.L. were drawing the figures. All authors read and approved the final version of the manuscript.

## Funding

This study was funded by the TaiShan Scholars of Shandong Province (No. tstp20221160) to Li M, the Construction Engineering Special Fund of “Taishan Young Scholars” of Shandong Province (No. tsqn202103177), the Natural Science Foundation of Shandong Province (No. ZR202210210042) to Liu H.

## Ethics Statement

The authors have nothing to report.

## Conflicts of Interest

The authors declare no conflicts of interest.

## Data Availability

The authors have nothing to report.

## References

[1] Q. Jiang , J. Liu , S. Huang , et al., “Antiageing Strategy for Neurodegenerative Diseases: From Mechanisms to Clinical Advances,” Signal Transduction and Targeted Therapy 10, no. 1 (2025): 76. 40059211 10.1038/s41392-025-02145-7 PMC11891338

[2] P. Scheltens , B. De Strooper , M. Kivipelto , et al., “Alzheimer's Disease,” Lancet 397, no. 10284 (2021): 1577–1590. 33667416 10.1016/S0140-6736(20)32205-4 PMC8354300

[3] M. M. Cláudio , V. G. Garcia , R. M. Freitas , et al., “Association of Active Oxygen‐Releasing Gel and Photodynamic Therapy in the Treatment of Residual Periodontal Pockets in Type 2 Diabetic Patients: A Randomized Controlled Clinical Study,” Journal of Periodontology 95, no. 4 (2023): 360–371. 38112075 10.1002/JPER.23-0125

[4] D. Öztürk , I. Midi , M. Alaylıoğlu , et al., “Serum and Saliva Biomolecules in Periodontitis Patients With and Without Alzheimer's Disease,” Nigerian Journal of Clinical Practice 28, no. 3 (2025): 409–416. 40214070 10.4103/njcp.njcp_254_24

[5] M. A. Beydoun , H. A. Beydoun , S. Hossain , Z. W. El‐Hajj , J. Weiss , and A. B. Zonderman , “Clinical and Bacterial Markers of Periodontitis and Their Association With Incident All‐Cause and Alzheimer's Disease Dementia in a Large National Survey,” Journal of Alzheimer's Disease 75, no. 1 (2020): 157–172. 10.3233/JAD-200064 PMC11008556 32280099

[6] A. Barbarisi , V. Visconti , D. Lauritano , F. Cremonini , G. Caccianiga , and S. Ceraulo , “Correlation Between Periodontitis and Onset of Alzheimer's Disease: A Literature Review,” Dentistry Journal (Basel) 12, no. 10 (2024): 331. 10.3390/dj12100331 PMC11505964 39452459

[7] S. J. Kittner and B. L. Taylor , “Oral Health and Brain Health: Cause, Consequence, or Confounding?,” Neurology 102, no. 6 (2024): e209251. 38165350 10.1212/WNL.0000000000208089

[8] S. M. Pruntel , B. C. van Munster , J. J. de Vries , A. Vissink , and A. Visser , “Oral Health as a Risk Factor for Alzheimer Disease,” Journal of Prevention of Alzheimer's Disease 11, no. 1 (2024): 249–258. 10.14283/jpad.2023.82 PMC10994994 38230738

[9] G. Hajishengallis and T. Chavakis , “Local and Systemic Mechanisms Linking Periodontal Disease and Inflammatory Comorbidities,” Nature Reviews. Immunology 21, no. 7 (2021): 426–440. 10.1038/s41577-020-00488-6 PMC7841384 33510490

[10] J. Wan and H. Fan , “Oral Microbiome and Alzheimer's Disease,” Microorganisms 11, no. 10 (2023): 2550. 37894208 10.3390/microorganisms11102550 PMC10609607

[11] A. Maldonado , O. Laugisch , W. Bürgin , A. Sculean , and S. Eick , “Clinical Periodontal Variables in Patients With and Without Dementia‐A Systematic Review and Meta‐Analysis,” Clinical Oral Investigations 22, no. 7 (2018): 2463–2474. 29934798 10.1007/s00784-018-2523-x

[12] B. Twarowski and M. Herbet , “Inflammatory Processes in Alzheimer's Disease‐Pathomechanism, Diagnosis and Treatment: A Review,” International Journal of Molecular Sciences 24, no. 7 (2023): 6518. 37047492 10.3390/ijms24076518 PMC10095343

[13] Z. Niu , X. Gui , S. Feng , and B. Reif , “Aggregation Mechanisms and Molecular Structures of Amyloid‐β in Alzheimer's Disease,” Chemistry 30, no. 48 (2024): e202400277. 38888453 10.1002/chem.202400277

[14] E. Cho , S. J. Jeon , J. Jeon , et al., “Phyllodulcin Improves Hippocampal Long‐Term Potentiation in 5XFAD Mice,” Biomedicine & Pharmacotherapy 161 (2023): 114511. 36913892 10.1016/j.biopha.2023.114511

[15] C. Parra Bravo , S. A. Naguib , and L. Gan , “Cellular and Pathological Functions of Tau,” Nature Reviews. Molecular Cell Biology 25, no. 11 (2024): 845–864. 39014245 10.1038/s41580-024-00753-9

[16] D. Giovinazzo , B. Bursac , J. I. Sbodio , et al., “Hydrogen Sulfide Is Neuroprotective in Alzheimer's Disease by Sulfhydrating GSK3β and Inhibiting Tau Hyperphosphorylation,” Proceedings of the National Academy of Sciences of the United States of America 118, no. 4 (2021): e2017225118. 33431651 10.1073/pnas.2017225118 PMC7848711

[17] C. Glynn , J. A. Rodriguez , and B. T. Hyman , “The Structural Line Between Prion and “Prion‐Like”: Insights From Prion Protein and Tau,” Current Opinion in Neurobiology 86 (2024): 102857. 38489865 10.1016/j.conb.2024.102857 PMC11162956

[18] V. Calsolaro and P. Edison , “Neuroinflammation in Alzheimer's Disease: Current Evidence and Future Directions,” Alzheimer's & Dementia 12, no. 6 (2016): 719–732. 10.1016/j.jalz.2016.02.010 27179961

[19] C. H. DeVore , J. E. Duckworth , F. M. Beck , M. J. Hicks , F. W. Brumfield , and J. E. Horton , “Bone Loss Following Periodontal Therapy in Subjects Without Frequent Periodontal Maintenance,” Journal of Periodontology 57, no. 6 (1986): 354–359. 3459858 10.1902/jop.1986.57.6.354

[20] N. S. Jakubovics , S. D. Goodman , L. Mashburn‐Warren , G. P. Stafford , and F. Cieplik , “The Dental Plaque Biofilm Matrix,” Periodontology 2000 86, no. 1 (2021): 32–56. 33690911 10.1111/prd.12361 PMC9413593

[21] S. Yang , Y. Yin , Y. Sun , et al., “AZGP1 Aggravates Macrophage M1 Polarization and Pyroptosis in Periodontitis,” Journal of Dental Research 103, no. 6 (2024): 631–641. 38491721 10.1177/00220345241235616

[22] B. Bassani , M. Cucchiara , A. Butera , et al., “Neutrophils' Contribution to Periodontitis and Periodontitis‐Associated Cardiovascular Diseases,” International Journal of Molecular Sciences 24, no. 20 (2023): 15370. 37895050 10.3390/ijms242015370 PMC10607037

[23] R. Gao , W. Zhang , Y. Jiang , et al., “Eldecalcitol Effectively Prevents Alveolar Bone Loss by Partially Improving Th17/Treg Cell Balance in Diabetes‐Associated Periodontitis,” Frontiers in Bioengineering and Biotechnology 11 (2023): 1070117. 36815882 10.3389/fbioe.2023.1070117 PMC9936814

[24] S. Li , S. Li , L. Meng , R. Gao , H. Liu , and M. Li , “Immunopathogenesis and Immunotherapy of Diabetes‐Associated Periodontitis,” Clinical Oral Investigations 29, no. 1 (2025): 44. 39755848 10.1007/s00784-024-06141-z

[25] F. R. M. Leite , G. G. Nascimento , F. Scheutz , and R. López , “Effect of Smoking on Periodontitis: A Systematic Review and Meta‐Regression,” American Journal of Preventive Medicine 54, no. 6 (2018): 831–841. 29656920 10.1016/j.amepre.2018.02.014

[26] V. Dibello , C. Custodero , R. Cavalcanti , et al., “Impact of Periodontal Disease on Cognitive Disorders, Dementia, and Depression: A Systematic Review and Meta‐Analysis,” Geroscience 46, no. 5 (2024): 5133–5169. 38943006 10.1007/s11357-024-01243-8 PMC11336026

[27] C.‐K. Chen , Y.‐T. Wu , and Y.‐C. Chang , “Association Between Chronic Periodontitis and the Risk of Alzheimer's Disease: A Retrospective, Population‐Based, Matched‐Cohort Study,” Alzheimer's Research & Therapy 9, no. 1 (2017): 56. 10.1186/s13195-017-0282-6 PMC5547465 28784164

[28] R. de Oliveira Araújo , G. E. M. Villoria , R. R. Luiz , J. C. Esteves , A. T. T. Leão , and E. J. Feres‐Filho , “Association Between Periodontitis and Alzheimer's Disease and Its Impact on the Self‐Perceived Oral Health Status: A Case‐Control Study,” Clinical Oral Investigations 25, no. 2 (2020): 555–562. 32772327 10.1007/s00784-020-03489-w

[29] S. Liu , S. G. Dashper , and R. Zhao , “Association Between Oral Bacteria and Alzheimer's Disease: A Systematic Review and Meta‐Analysis,” Journal of Alzheimer's Disease 91, no. 1 (2023): 129–150. 10.3233/JAD-220627 36404545

[30] M. A. Beydoun , H. A. Beydoun , D. W. Hedges , et al., “Infection Burden, Periodontal Pathogens, and Their Interactive Association With Incident All‐Cause and Alzheimer's Disease Dementia in a Large National Survey,” Alzheimer's & Dementia 20, no. 9 (2024): 6468–6485. 10.1002/alz.14141 PMC11497652 39115027

[31] S. Mao , C.‐P. Huang , H. Lan , H.‐G. Lau , C.‐P. Chiang , and Y.‐W. Chen , “Association of Periodontitis and Oral Microbiomes With Alzheimer's Disease: A Narrative Systematic Review,” Journal of Dental Sciences 17, no. 4 (2022): 1762–1779. 36299333 10.1016/j.jds.2022.07.001 PMC9588805

[32] H. Larvin , C. Gao , J. Kang , V. R. Aggarwal , S. Pavitt , and J. Wu , “The Impact of Study Factors in the Association of Periodontal Disease and Cognitive Disorders: Systematic Review and Meta‐Analysis,” Age and Ageing 52, no. 2 (2023): afad015. 36794714 10.1093/ageing/afad015 PMC10789237

[33] Y. D. Fu , C. L. Li , C. L. Hu , et al., “Meta Analysis of the Correlation Between Periodontal Health and Cognitive Impairment in the Older Population,” Journal of Prevention of Alzheimer's Disease 11, no. 5 (2024): 1307–1315. 10.14283/jpad.2024.87 39350376

[34] S. Choi , K. Kim , J. Chang , et al., “Association of Chronic Periodontitis on Alzheimer's Disease or Vascular Dementia,” Journal of the American Geriatrics Society 67, no. 6 (2019): 1234–1239. 30874308 10.1111/jgs.15828

[35] K. Karaduran , A. Aydogdu , O. Gelisin , and S. Gunpinar , “Investigating the Potential Clinical Impact of Periodontitis on the Progression of Alzheimer's Disease: A Prospective Cohort Study,” Clinical Oral Investigations 28, no. 1 (2023): 67. 38159159 10.1007/s00784-023-05445-w

[36] C. Schwahn , S. Frenzel , B. Holtfreter , et al., “Effect of Periodontal Treatment on Preclinical Alzheimer's Disease‐Results of a Trial Emulation Approach,” Alzheimer's & Dementia 18, no. 1 (2021): 127–141. 10.1002/alz.12378 34050719

[37] C. Marruganti , G. Baima , M. Aimetti , S. Grandini , M. Sanz , and M. Romandini , “Periodontitis and Low Cognitive Performance: A Population‐Based Study,” Journal of Clinical Periodontology 50, no. 4 (2023): 418–429. 36644802 10.1111/jcpe.13779

[38] K. S. Ma , H. Hasturk , I. Carreras , et al., “Dementia and the Risk of Periodontitis: A Population‐Based Cohort Study,” Journal of Dental Research 101, no. 3 (2021): 270–277. 34643147 10.1177/00220345211037220 PMC8982009

[39] Z. Tang , X. Cheng , X. Su , L. Wu , Q. Cai , and H. Wu , “ *Treponema denticola* Induces Alzheimer‐Like Tau Hyperphosphorylation by Activating Hippocampal Neuroinflammation in Mice,” Journal of Dental Research 101, no. 8 (2022): 992–1001. 35193423 10.1177/00220345221076772

[40] G. D. Ciccotosto , A. I. Mohammed , R. Paolini , et al., “Chronic Oral Inoculation of Porphyromonas Gingivalis and *Treponema denticola* Induce Different Brain Pathologies in a Mouse Model of Alzheimer Disease,” Journal of Infectious Diseases 230, no. Supplement_2 (2024): S109–S116. 39255392 10.1093/infdis/jiae286

[41] X. Li , M. Kiprowska , T. Kansara , P. Kansara , and P. Li , “Neuroinflammation: A Distal Consequence of Periodontitis,” Journal of Dental Research 101, no. 12 (2022): 1441–1449. 35708472 10.1177/00220345221102084 PMC9608094

[42] S. Lei , J. Li , J. Yu , et al., “ *Porphyromonas gingivalis* Bacteremia Increases the Permeability of the Blood‐Brain Barrier via the Mfsd2a/Caveolin‐1 Mediated Transcytosis Pathway,” International Journal of Oral Science 15, no. 1 (2023): 3. 36631446 10.1038/s41368-022-00215-y PMC9834243

[43] S. Nonaka , T. Kadowaki , and H. Nakanishi , “Secreted Gingipains From *Porphyromonas gingivalis* Increase Permeability in Human Cerebral Microvascular Endothelial Cells Through Intracellular Degradation of Tight Junction Proteins,” Neurochemistry International 154 (2022): 105282. 35032577 10.1016/j.neuint.2022.105282

[44] N. Sato , T. Matsumoto , S. Kawaguchi , et al., “ *Porphyromonas gingivalis* Lipopolysaccharide Induces Interleukin‐6 and c‐c Motif Chemokine Ligand 2 Expression in Cultured hCMEC/D3 Human Brain Microvascular Endothelial Cells,” Gerodontology 39, no. 2 (2021): 139–147. 33599317 10.1111/ger.12545

[45] M. Elashiry , A. Carroll , J. Yuan , et al., “Oral Microbially‐Induced Small Extracellular Vesicles Cross the Blood‐Brain Barrier,” International Journal of Molecular Sciences 25, no. 8 (2024): 4509. 38674094 10.3390/ijms25084509 PMC11049816

[46] Y. Zhang , Y. Sun , Y. Hu , et al., “ *Porphyromonas gingivalis* msRNA P.G_45033 Induces Amyloid‐β Production by Enhancing Glycolysis and Histone Lactylation in Macrophages,” International Immunopharmacology 121 (2023): 110468. 37320870 10.1016/j.intimp.2023.110468

[47] X. Ma , Y.‐J. Shin , J.‐W. Yoo , H.‐S. Park , and D.‐H. Kim , “Extracellular Vesicles Derived From *Porphyromonas gingivalis* Induce Trigeminal Nerve‐Mediated Cognitive Impairment,” Journal of Advanced Research 54 (2023): 293–303. 36796586 10.1016/j.jare.2023.02.006 PMC10703712

[48] J. Lu , S. Zhang , Y. Huang , et al., “Periodontitis‐Related Salivary Microbiota Aggravates Alzheimer's Disease via Gut‐Brain Axis Crosstalk,” Gut Microbes 14, no. 1 (2022): 2126272. 36175166 10.1080/19490976.2022.2126272 PMC9542625

[49] K. A. Ravichandran and M. T. Heneka , “Inflammasomes in Neurological Disorders – Mechanisms and Therapeutic Potential,” Nature Reviews. Neurology 20, no. 2 (2024): 67–83. 38195712 10.1038/s41582-023-00915-x

[50] X. Zhang , X. Zhang , C. Qiu , et al., “The Imbalance of Th17/Treg via STAT3 Activation Modulates Cognitive Impairment in *P. gingivalis* LPS‐Induced Periodontitis Mice,” Journal of Leukocyte Biology 110, no. 3 (2021): 511–524. 34342041 10.1002/JLB.3MA0521-742RRR

[51] J. M. Lawrence , K. Schardien , B. Wigdahl , and M. R. Nonnemacher , “Roles of Neuropathology‐Associated Reactive Astrocytes: A Systematic Review,” Acta Neuropathologica Communications 11, no. 1 (2023): 42. 36915214 10.1186/s40478-023-01526-9 PMC10009953

[52] A. M. Chaple‐Gil , M. Santiesteban‐Velázquez , and J. J. Urbizo Vélez , “Association Between Oral Microbiota Dysbiosis and the Risk of Dementia: A Systematic Review,” Dentistry Journal (Basel) 13, no. 6 (2025): 227. 10.3390/dj13060227 PMC12191743 40559130

[53] A. Sharma , S. Inagaki , K. Honma , C. Sfintescu , P. J. Baker , and R. T. Evans , “ *Tannerella forsythia*‐Induced Alveolar Bone Loss in Mice Involves Leucine‐Rich‐Repeat BspA Protein,” Journal of Dental Research 84, no. 5 (2005): 462–467. 15840784 10.1177/154405910508400512

[54] Y. Lim , H. Y. Kim , D. Han , and B.‐K. Choi , “Proteome and Immune Responses of Extracellular Vesicles Derived From Macrophages Infected With the Periodontal Pathogen *Tannerella forsythia* ,” Journal of Extracellular Vesicles 12, no. 12 (2023): e12381. 38014595 10.1002/jev2.12381 PMC10682907

[55] G. Hajishengallis , M. Martin , H. T. Sojar , et al., “Dependence of Bacterial Protein Adhesins on Toll‐Like Receptors for Proinflammatory Cytokine Induction,” Clinical and Diagnostic Laboratory Immunology 9, no. 2 (2002): 403–411. 11874886 10.1128/CDLI.9.2.403-411.2002 PMC119939

[56] M. Yoneda , T. Yoshikane , N. Motooka , et al., “Stimulation of Growth of *Porphyromonas gingivalis* by Cell Extracts From *Tannerella forsythia* ,” Journal of Periodontal Research 40, no. 2 (2005): 105–109. 15733144 10.1111/j.1600-0765.2005.00774.x

[57] K. Saiki , Y. Urano‐Tashiro , Y. Yamanaka , and Y. Takahashi , “Calcium Ions and Vitamin B12 Are Growth Factors for *Porphyromonas gingivalis* ,” Journal of Oral Biosciences 64, no. 4 (2022): 445–451. 36103977 10.1016/j.job.2022.09.001

[58] A. K. McGregor , A. C. K. Chan , M. D. Schroeder , et al., “A New Member of the Flavodoxin Superfamily From *Fusobacterium nucleatum* That Functions in Heme Trafficking and Reduction of Anaerobilin,” Journal of Biological Chemistry 299, no. 7 (2023): 104902. 37302554 10.1016/j.jbc.2023.104902 PMC10404700

[59] P. M. Jansen , M. M. H. Abdelbary , and G. Conrads , “A Concerted Probiotic Activity to Inhibit Periodontitis‐Associated Bacteria,” PLoS One 16, no. 3 (2021): e0248308. 33667279 10.1371/journal.pone.0248308 PMC7935250

[60] W. C. Pierre , I. Londono , C. Quiniou , S. Chemtob , and G. A. Lodygensky , “Modulatory Effect of IL‐1 Inhibition Following Lipopolysaccharide‐Induced Neuroinflammation in Neonatal Microglia and Astrocytes,” International Journal of Developmental Neuroscience 82, no. 3 (2022): 243–260. 35315121 10.1002/jdn.10179

[61] T. Gong , Q. Chen , H. Mao , et al., “Outer Membrane Vesicles of *Porphyromonas gingivalis* Trigger NLRP3 Inflammasome and Induce Neuroinflammation, Tau Phosphorylation, and Memory Dysfunction in Mice,” Frontiers in Cellular and Infection Microbiology 12 (2022): 925435. 36017373 10.3389/fcimb.2022.925435 PMC9397999

[62] M. Jiang , X. Zhang , X. Yan , et al., “GSK3β Is Involved in Promoting Alzheimer's Disease Pathologies Following Chronic Systemic Exposure to *Porphyromonas gingivalis* Lipopolysaccharide in Amyloid Precursor proteinNL‐F/NL‐F Knock‐In Mice,” Brain, Behavior, and Immunity 98 (2021): 1–12. 34391814 10.1016/j.bbi.2021.08.012 PMC8849844

[63] B. Wu , M. Chen , L. Meng , Q. Tian , and Z. Dong , “Osteoclasts Link Dysregulated Peripheral Degradation Processes and Accelerated Progression in Alzheimer's Disease,” Journal of Alzheimer's Disease 99, no. 2 (2024): 773–785. 10.3233/JAD-240096 38701149

[64] Y. Weng , H. Wang , L. Li , Y. Feng , S. Xu , and Z. Wang , “Trem2 Mediated Syk‐Dependent ROS Amplification Is Essential for Osteoclastogenesis in Periodontitis Microenvironment,” Redox Biology 40 (2020): 101849. 33486152 10.1016/j.redox.2020.101849 PMC7823053

[65] C. Arévalo‐Caro , M. Arce Retana , S. Losada Amaya , H. Arboleda , X. Gallart‐Palau , and A. Serra , “APOE4, Alzheimer's and Periodontal Disease: A Scoping Review,” Ageing Research Reviews 105 (2025): 102649. 39864561 10.1016/j.arr.2024.102649

[66] S. S. Dominy , C. Lynch , F. Ermini , et al., “ *Porphyromonas gingivalis* in Alzheimer's Disease Brains: Evidence for Disease Causation and Treatment With Small‐Molecule Inhibitors,” Science Advances 5, no. 1 (2019): eaau3333. 30746447 10.1126/sciadv.aau3333 PMC6357742

[67] R. Jin , X. Ning , X. Liu , Y. Zhao , and G. Ye , “ *Porphyromonas gingivalis*‐Induced Periodontitis Could Contribute to Cognitive Impairment in Sprague‐Dawley Rats via the P38 MAPK Signaling Pathway,” Frontiers in Cellular Neuroscience 17 (2023): 1141339. 37056710 10.3389/fncel.2023.1141339 PMC10086325

[68] H. Wu , W. Qiu , X. Zhu , et al., “The Periodontal Pathogen *Fusobacterium nucleatum* Exacerbates Alzheimer's Pathogenesis via Specific Pathways,” Frontiers in Aging Neuroscience 14 (2022): 912709. 35813949 10.3389/fnagi.2022.912709 PMC9260256

[69] B. Kalyanaraman , G. Cheng , and M. Hardy , “Gut Microbiome, Short‐Chain Fatty Acids, Alpha‐Synuclein, Neuroinflammation, and ROS/RNS: Relevance to Parkinson's Disease and Therapeutic Implications,” Redox Biology 71 (2024): 103092. 38377788 10.1016/j.redox.2024.103092 PMC10891329

[70] H. Chen , Y. Liao , X. Zhang , et al., “Age‐ and Sex‐Related Differences of Periodontal Bone Resorption, Cognitive Function, and Immune State in APP/PS1 Murine Model of Alzheimer's Disease,” Journal of Neuroinflammation 20, no. 1 (2023): 153. 37370108 10.1186/s12974-023-02790-1 PMC10294321

[71] C. Xie , C. Wang , and H. Luo , “Increased Risk of Osteoporosis in Patients With Cognitive Impairment: A Systematic Review and Meta‐Analysis,” BMC Geriatrics 23, no. 1 (2023): 797. 38049723 10.1186/s12877-023-04548-z PMC10694915

[72] S. Cui , F. Xiong , Y. Hong , et al., “APPswe/Aβ Regulation of Osteoclast Activation and RAGE Expression in an Age‐Dependent Manner,” Journal of Bone and Mineral Research 26, no. 5 (2011): 1084–1098. 21542009 10.1002/jbmr.299 PMC3126661

[73] S. Li , B. Yang , D. Teguh , L. Zhou , J. Xu , and L. Rong , “Amyloid β Peptide Enhances RANKL‐Induced Osteoclast Activation Through NF‐κB, ERK, and Calcium Oscillation Signaling,” International Journal of Molecular Sciences 17, no. 10 (2016): 1683. 27735865 10.3390/ijms17101683 PMC5085715

[74] N. M. Trang , E.‐N. Kim , T. H. Pham , and G.‐S. Jeong , “Citropten Ameliorates Osteoclastogenesis Related to MAPK and PLCγ/Ca2+ Signaling Pathways Through the Regulation of Amyloid Beta,” Journal of Agricultural and Food Chemistry 71, no. 26 (2023): 10037–10049. 37260315 10.1021/acs.jafc.3c00368

[75] W.‐F. Xia , J.‐U. Jung , C. Shun , et al., “Swedish Mutant APP Suppresses Osteoblast Differentiation and Causes Osteoporotic Deficit, Which Are Ameliorated by N‐Acetyl‐L‐Cysteine,” Journal of Bone and Mineral Research 28, no. 10 (2013): 2122–2135. 23649480 10.1002/jbmr.1954 PMC7104794

[76] W. Zhang , F. Ding , X. Rong , et al., “Aβ ‐Induced Excessive Mitochondrial Fission Drives Type H Blood Vessels Injury to Aggravate Bone Loss in APP/PS1 Mice With Alzheimer's Diseases,” Aging Cell 24, no. 2 (2024): e14374. 39411913 10.1111/acel.14374 PMC11822656

[77] Q. Guo , N. Chen , C. Qian , et al., “Sympathetic Innervation Regulates Osteocyte‐Mediated Cortical Bone Resorption During Lactation,” Advanced Science (Weinh) 10, no. 18 (2023): e2207602. 10.1002/advs.202207602 PMC10288263 37186379

[78] Y. Okada , N. Hamada , Y. Kim , et al., “Blockade of Sympathetic b‐Receptors Inhibits *Porphyromonas gingivalis* ‐Induced Alveolar Bone Loss in an Experimental Rat Periodontitis Model,” Archives of Oral Biology 55, no. 7 (2010): 502–508. 20593554 10.1016/j.archoralbio.2010.04.002

[79] F. Elefteriou , “Impact of the Autonomic Nervous System on the Skeleton,” Physiological Reviews 98, no. 3 (2018): 1083–1112. 29717928 10.1152/physrev.00014.2017 PMC6088147

[80] F. Elefteriou , J. D. Ahn , S. Takeda , et al., “Leptin Regulation of Bone Resorption by the Sympathetic Nervous System and CART,” Nature 434, no. 7032 (2005): 514–520. 15724149 10.1038/nature03398

[81] H. Kondo , M. Kondo , K. Hayashi , et al., “Orthodontic Tooth Movement‐Activated Sensory Neurons Contribute to Enhancing Osteoclast Activity and Tooth Movement Through Sympathetic Nervous Signalling,” European Journal of Orthodontics 44, no. 4 (2022): 404–411. 34642757 10.1093/ejo/cjab072

[82] D. Geng , Y. Wang , Z. Gao , J. Wang , X. Liu , and G. Pang , “Effects of Alzheimer's Disease of Varying Severity on Cardiac and Autonomic Function,” Brazilian Journal of Medical and Biological Research 55 (2022): e11504. 35019033 10.1590/1414-431X2021e11504 PMC8851908

[83] P. Sharan and C. Vellapandian , “Hypothalamic‐Pituitary‐Adrenal (HPA) Axis: Unveiling the Potential Mechanisms Involved in Stress‐Induced Alzheimer's Disease and Depression,” Cureus 16, no. 8 (2024): e67595. 39310640 10.7759/cureus.67595 PMC11416836

[84] Y. Zhao , X. Peng , Q. Wang , et al., “Crosstalk Between the Neuroendocrine System and Bone Homeostasis,” Endocrine Reviews 45, no. 1 (2024): 95–124. 37459436 10.1210/endrev/bnad025

[85] G. K. Banoriya , V. K. Singh , R. Maurya , and R. K. Kharwar , “Neuro‐Immuno‐Endocrine Regulation of Bone Homeostasis,” Discovery Medicine 37, no. 194 (2025): 464–485. 40116095 10.24976/Discov.Med.202537194.39

[86] G. Muscogiuri , B. Altieri , M. Penna‐Martinez , and K. Badenhoop , “Focus on Vitamin D and the Adrenal Gland,” Hormone and Metabolic Research 47, no. 4 (2015): 239–246. 25723858 10.1055/s-0034-1396893

[87] S. J. Karnik , T. J. Margetts , H. S. Wang , et al., “Mind the Gap: Unraveling the Intricate Dance Between Alzheimer's Disease and Related Dementias and Bone Health,” Current Osteoporosis Reports 22, no. 1 (2024): 165–176. 38285083 10.1007/s11914-023-00847-x PMC10912190

[88] Y. Peng , S.‐Y. Yao , Q. Chen , et al., “True or False? Alzheimer's Disease Is Type 3 Diabetes: Evidences From Bench to Bedside,” Ageing Research Reviews 99 (2024): 102383. 38955264 10.1016/j.arr.2024.102383

[89] A. Salehipour , M. Dolatshahi , M. Haghshomar , and J. Amin , “The Role of Thyroid Dysfunction in Alzheimer's Disease: A Systematic Review and Meta‐Analysis,” Journal of Prevention of Alzheimer's Disease 10, no. 2 (2023): 276–286. 10.14283/jpad.2023.20 36946455

[90] G. Pfister , D. Weiskopf , L. Lazuardi , et al., “Naive T Cells in the Elderly: Are They Still There?,” Annals of the New York Academy of Sciences 1067 (2006): 152–157. 16803980 10.1196/annals.1354.018

[91] G. Monasterio , B. Fernández , F. Castillo , et al., “Capsular‐Defective *Porphyromonas gingivalis* Mutant Strains Induce Less Alveolar Bone Resorption Than W50 Wild‐Type Strain due to a Decreased Th1/Th17 Immune Response and Less Osteoclast Activity,” Journal of Periodontology 90, no. 5 (2018): 522–534. 30397909 10.1002/JPER.18-0079

[92] M. Jorfi , A. Maaser‐Hecker , and R. E. Tanzi , “The Neuroimmune Axis of Alzheimer's Disease,” Genome Medicine 15, no. 1 (2023): 6. 36703235 10.1186/s13073-023-01155-w PMC9878767

[93] Z. Wang , A. Saxena , W. Yan , S. M. Uriarte , R. Siqueira , and X. Li , “The Impact of Aging on Neutrophil Functions and the Contribution to Periodontitis,” International Journal of Oral Science 17, no. 1 (2025): 10. 39819982 10.1038/s41368-024-00332-w PMC11739572

[94] Q. Zhang , G. Yang , Y. Luo , L. Jiang , H. Chi , and G. Tian , “Neuroinflammation in Alzheimer's Disease: Insights From Peripheral Immune Cells,” Immunity & Ageing 21, no. 1 (2024): 38. 38877498 10.1186/s12979-024-00445-0 PMC11177389

[95] A. Ciaramella , F. Bizzoni , F. Salani , et al., “Increased Pro‐Inflammatory Response by Dendritic Cells From Patients With Alzheimer's Disease,” Journal of Alzheimer's Disease 19, no. 2 (2010): 559–572. 10.3233/JAD-2010-1257 20110602

[96] A. Wilensky , H. Segev , G. Mizraji , et al., “Dendritic Cells and Their Role in Periodontal Disease,” Oral Diseases 20, no. 2 (2013): 119–126. 23656605 10.1111/odi.12122

[97] P. Bossù , G. Spalletta , C. Caltagirone , and A. Ciaramella , “Myeloid Dendritic Cells Are Potential Players in Human Neurodegenerative Diseases,” Frontiers in Immunology 6 (2015): 632. 26734003 10.3389/fimmu.2015.00632 PMC4679857

[98] Y. L. Kapila , “Oral Health's Inextricable Connection to Systemic Health: Special Populations Bring to Bear Multimodal Relationships and Factors Connecting Periodontal Disease to Systemic Diseases and Conditions,” Periodontology 2000 87, no. 1 (2021): 11–16. 34463994 10.1111/prd.12398 PMC8457130

[99] A. A. Belaidi , A. I. Bush , and S. Ayton , “Apolipoprotein E in Alzheimer's Disease: Molecular Insights and Therapeutic Opportunities,” Molecular Neurodegeneration 20, no. 1 (2025): 47. 40275327 10.1186/s13024-025-00843-y PMC12023563

[100] G. Malcangi , G. Marinelli , A. D. Inchingolo , et al., “Salivaomics: New Frontiers in Studying the Relationship Between Periodontal Disease and Alzheimer's Disease,” Metabolites 15, no. 6 (2025): 389. 40559413 10.3390/metabo15060389 PMC12195213

[101] J. Chen , H. Chen , Q. Wei , et al., “APOE4 Impairs Macrophage Lipophagy and Promotes Demyelination of Spiral Ganglion Neurons in Mouse Cochleae,” Cell Death Discovery 11, no. 1 (2025): 190. 40258814 10.1038/s41420-025-02454-4 PMC12012174

[102] L. Arnaud , P. Benech , L. Greetham , et al., “APOE4 Drives Inflammation in Human Astrocytes via TAGLN3 Repression and NF‐κB Activation,” Cell Reports 40, no. 7 (2022): 111200. 35977506 10.1016/j.celrep.2022.111200

[103] M. M. Peshoff , P. Gupta , S. Oberai , et al., “Triggering Receptor Expressed on Myeloid Cells 2 (TREM2) Regulates Phagocytosis in Glioblastoma,” Neuro‐Oncology 26, no. 5 (2024): 826–839. 38237157 10.1093/neuonc/noad257 PMC11066944

[104] T. K. Ulland , W. M. Song , S. C.‐C. Huang , et al., “TREM2 Maintains Microglial Metabolic Fitness in Alzheimer's Disease,” Cell 170, no. 4 (2017): 649–663.e13. 28802038 10.1016/j.cell.2017.07.023 PMC5573224

[105] M. H. A. Saleh and H. Sabri , “Dose‐Dependent Association of Systemic Comorbidities With Periodontitis Severity: A Large Population Cross‐Sectional Study,” Journal of Periodontology (2025): 1–16. 10.1002/JPER.25-0055 PMC13001135 40778524

[106] R. A. V. Lacerda , J. A. F. Desio , C. M. Kammers , et al., “Sleep Disorders and Risk of Alzheimer's Disease: A Two‐Way Road,” Ageing Research Reviews 101 (2024): 102514. 39317268 10.1016/j.arr.2024.102514

[107] L. Chen , W. Nini , Z. Jinmei , and Y. Jingmei , “Implications of Sleep Disorders for Periodontitis,” Sleep and Breathing 27, no. 5 (2022): 1655–1666. 36547852 10.1007/s11325-022-02769-x

[108] D. Herrera , M. Sanz , L. Shapira , et al., “Association Between Periodontal Diseases and Cardiovascular Diseases, Diabetes and Respiratory Diseases: Consensus Report of the Joint Workshop by the European Federation of Periodontology (EFP) and the European Arm of the World Organization of Family Doctors (WONCA Europe),” Journal of Clinical Periodontology 50, no. 6 (2023): 819–841. 36935200 10.1111/jcpe.13807

[109] S. E. Arnold , Z. Arvanitakis , S. L. Macauley‐Rambach , et al., “Brain Insulin Resistance in Type 2 Diabetes and Alzheimer Disease: Concepts and Conundrums,” Nature Reviews. Neurology 14, no. 3 (2018): 168–181. 29377010 10.1038/nrneurol.2017.185 PMC6098968

[110] A. Saeed , O. Lopez , A. Cohen , and S. E. Reis , “Cardiovascular Disease and Alzheimer's Disease: The Heart‐Brain Axis,” Journal of the American Heart Association 12, no. 21 (2023): e030780. 37929715 10.1161/JAHA.123.030780 PMC10727398

[111] G. Tondo , F. De Marchi , E. Terazzi , et al., “Chronic Obstructive Pulmonary Disease May Complicate Alzheimer's Disease: A Comorbidity Problem,” Neurological Sciences 39, no. 9 (2018): 1585–1589. 29931515 10.1007/s10072-018-3470-7

[112] M. Oray , K. Abu Samra , N. Ebrahimiadib , H. Meese , and C. S. Foster , “Long‐Term Side Effects of Glucocorticoids,” Expert Opinion on Drug Safety 15, no. 4 (2016): 457–465. 26789102 10.1517/14740338.2016.1140743

[113] P. S. Farsai , “Cognitive Impairment in Older Adults and Oral Health Considerations: Treatment and Management,” Clinics in Geriatric Medicine 39, no. 2 (2023): 295–310. 37045534 10.1016/j.cger.2023.01.001

[114] S. S. Gao , C. H. Chu , and F. Y. F. Young , “Integrating 5S Methodology Into Oral Hygiene Practice for Elderly With Alzheimer's Disease,” Dentistry Journal (Basel) 8, no. 2 (2020): 29. 10.3390/dj8020029 PMC7344890 32225090

[115] C. H. Campos , G. R. Ribeiro , and R. C. M. Rodrigues Garcia , “Mastication and Oral Health‐Related Quality of Life in Removable Denture Wearers With Alzheimer Disease,” Journal of Prosthetic Dentistry 119, no. 5 (2017): 764–768. 28967408 10.1016/j.prosdent.2017.07.010

[116] S. A. Hamza , S. Asif , and S. A. H. Bokhari , “Oral Health of Individuals With Dementia and Alzheimer's Disease: A Review,” Journal of Indian Society of Periodontology 25, no. 2 (2021): 96. 33888939 10.4103/jisp.jisp_287_20 PMC8041071

[117] T. Kusama , K. Takeuchi , S. Kiuchi , J. Aida , and K. Osaka , “Poor Oral Health and Dementia Risk Under Time‐Varying Confounding: A Cohort Study Based on Marginal Structural Models,” Journal of the American Geriatrics Society 72, no. 3 (2023): 729–741. 38064294 10.1111/jgs.18707

[118] S. Suma , M. Furuta , K. Takeuchi , M. Tomioka , Y. Iwasa , and Y. Yamashita , “Number of Teeth, Denture Wearing and Cognitive Function in Relation to Nutritional Status in Residents of Nursing Homes,” Gerodontology 39, no. 2 (2021): 197–203. 34047382 10.1111/ger.12554

[119] T. Kim , S. I. Chi , H. Kim , and K.‐S. Seo , “Analysis of Behavioral Management for Dental Treatment in Patients With Dementia Using the Korean National Health Insurance Data,” Journal of Dental Anesthesia and Pain Medicine 21, no. 5 (2021): 461–469. 34703895 10.17245/jdapm.2021.21.5.461 PMC8520838

